# Histone modifications in the regulation of erythropoiesis

**DOI:** 10.1080/07853890.2025.2490824

**Published:** 2025-04-11

**Authors:** Xiuyun Wu, Hongdi Xu, Erxi Xia, Linru Gao, Yan Hou, Lei Sun, Hengchao Zhang, Ying Cheng

**Affiliations:** School of Life Sciences, Zhengzhou University, Zhengzhou, China

**Keywords:** Erythropoiesis, early hematopoiesis, histone methylation, histone acetylation, gene regulation

## Abstract

**Introduction:**

The pathogenesis of anemia and other erythroid dysphasia are mains poorly understood, primarily due to limited knowledge about the differentiation processes and regulatory mechanisms governing erythropoiesis. Erythropoiesis is a highly complex and precise biological process, that can be categorized into three distinct stages: early erythropoiesis, terminal erythroid differentiation, and reticulocyte maturation, and this complex process is tightly controlled by multiple regulatory factors. Emerging evidence highlights the crucial role of epigenetic modifications, particularly histone modifications, in regulating erythropoiesis. Methylation and acetylation are two common modification forms that affect genome accessibility by altering the state of chromatin, thereby regulating gene expression during erythropoiesis.

**Discussion:**

This review systematically examines the roles of histone methylation and acetylation, along with their respective regulatory enzymes, in regulating the development and differentiation of hematopoietic stem/progenitor cells (HSPCs) and erythroid progenitors. Furthermore, we discuss the involvement of these histone modifications in erythroid-specific developmental processes, including hemoglobin switching, chromatin condensation, and enucleation.

**Conclusions **

This review summarizes the current understanding of the role of histone modifications in erythropoiesis based on existing research, as a foundation for further research the mechanisms of epigenetic regulatory in erythropoiesis.

## PLAIN LANGUAGE SUMMARY

This review comprehensively examines the regulatory roles of histone methylation and acetylation in erythropoiesis, which is a complex and precise biological process including the early stage of erythroid development, terminal erythroid differentiation and the stage of red blood cells maturation. This article reviews histone modifications, mainly the regulatory effects of histone methylation and acetylation on early hematopoiesis and erythropoiesis, emphasizing the overlapping and unique roles of different histone modification modes in ensuring normal red blood cell development.

## 1. Introduction

Erythropoiesis represents a tightly regulated developmental process through which hematopoietic stem cells (HSCs) in the bone marrow undergo progressive differentiation to generate mature red blood cells (RBCs). This complex biological process can be conceptually divided into three major phases: (1) The early hematopoietic stem cell commitment stage: The erythropoietic cascade initiates with the differentiation of multipotent HSCs into bipotent megakaryocyte-erythrocyte progenitors (MEPs) in the bone marrow. HSCs can proliferate and differentiate in response to specific internal and external signaling stimuli. Among them, the regulation of erythropoiesis is mainly controlled by a hormone called erythropoietin (EPO). (2) Directed differentiation stage: MEPs continue to differentiate into early erythroid progenitor cells, including burst forming unit-erythroid cells (BFU-Es) and colony-forming unit-erythroid cells (CFU-Es). These erythroid progenitors possess limited self-renewal capabilities and a robust differentiation potential, during which specific genes indicative of erythropoiesis are gradually expressed [1]. (3) Terminal erythroid differentiation: proerythroblasts (Pro-Es) differentiated from CFU-Es continue to differentiate downward, which is the beginning of terminal differentiation. Pro-Es differentiate into erythroid precursors including basophilic erythroblasts (Baso-Es), polychromatic erythroblasts (Poly-Es), and orthochromatic erythroblasts (Ortho-Es). In this stage, some erythroid differentiation-specific events occur, such as decreased cell size, chromatin condensation, hemoglobin accumulation, and enucleation. Finally, Ortho-Es removes the nucleus to form reticulocytes, which further abandon other organelles include mitochondrial and ribosomal to become mature erythrocytes [2]. Eventually, red blood cells release bone marrow into the bloodstream and are delivered throughout the body for oxygen and carbon dioxide transport [1,3,4]. Numerous growth factors, hormones, and signaling pathways are required for regulation throughout erythropoiesis.

DNA is structured into chromatin by being coiled around histone proteins to constitute nucleosomes, which serve as the fundamental structural units of chromatin. Chromatin manifests in two primary forms: euchromatin, which is relatively uncondensed and transcriptionally active, and heterochromatin, which is highly condensed and linked to gene silencing. Heterochromatin encompasses constitutive regions (such as centromeres and telomeres) as well as maintaining genomic stability and controlling cell differentiation [5,6]. Beneath the nuclear lamina in most mammalian cells lies a dense layer of heterochromatin, referred to as the lamina-associated domains (LADs). These LADs are enriched with repressive histone modifications such as H3K9me2, H3K9me3, and H3K27me3 [7,8]. Proper deposition of heterochromatin is crucial for the packaging of the genome, ensuring appropriate gene silencing. Chromatin condensation and enucleation are characteristic biological events that occur at the terminal stage of erythropoiesis. Previous studies have found that histone-modifying enzymes, by mediating various histone modifications and collectively forming a ‘histone code’, maintain chromatin structure and dynamic changes, playing a crucial regulatory role in chromatin condensation and enucleation [9].

Erythropoiesis is a finely regulated process that involves multiple stages of differentiation and maturation, as well as changes in gene expression, chromatin structure, and cellular morphology. Epigenetic modifications, such as DNA methylation, histone modifications, and non-coding RNAs, play important roles in erythropoiesis by modulating the accessibility and activity of erythroid-specific genes and transcription factors [10–13]. In this review, we summarize the current knowledge on the epigenetic landscape of erythropoiesis, focusing on the changes in histone methylation and acetylation during different stages of erythroid differentiation, from HSCs to mature RBCs. Although, more research has focused on the function of histone methylation and acetylation, and the modification regulators in hematopoietic stem progenitor cells stage, we also discuss how epigenetic modifications affect specific events in erythropoiesis, such as hemoglobin switching, chromatin condensation, and enucleation.

## 2. Methylation modification of histone

**Figure 1.**
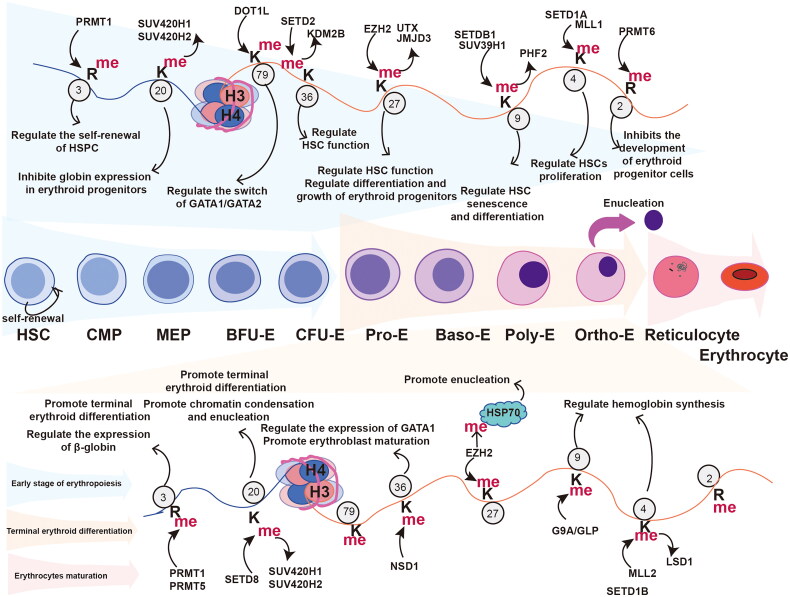
The role of histone methylation in erythropoiesis. Erythropoiesis is the process of HSC differentiation into erythrocyte, which is mainly divided into three stages: the early erythropoiesis stage (light blue differentiation path), terminal erythroid differentiation stage (light orange differentiation path), and erythrocyte maturation stage (pink path). The upper part of the diagram shows the role of histone methylation and related modifying enzymes (numbers representing methylation sites, R representing arginine residues, K representing lysine residues, protein-directed methylation indicating that the protein is methyltransferases, and methylation-directed protein indicating that the protein is demethylase) in early erythropoiesis. It is mainly involved in regulating the function of hematopoietic stem progenitor cells and the growth and development of erythroid progenitor cells. The lower part of the diagram shows the role of histone methylation and related modification enzymes in the terminal erythroid differentiation, which mainly regulates chromatin condensation, enucleation and globin expression in erythroblast.

Histone methylation can regulate gene expression by changing the structure of chromatin and is closely related to DNA methylation. This modification usually occurs on the lysine residues and arginine residues of histone H3 or H4 [14]. There are three methylation states of histone lysine residues: monomethylation, dimethylation and trimethylation [15]. The methylation state of histone arginine residues includes monomethylation (me1), symmetrically demethylation (me2s), or asymemetrically demethylation (me2a), depending on the location of methylation [16]. The dynamic regulation of histone methylation is mediated by opposing enzymatic activities of methyltransferases and demethylases, which respectively catalyze the addition and removal of methyl groups on specific amino acid residues. These modifications establish distinct chromatin states, including transcriptionally active (euchromatic), silent (heterochromatic), and bivalent domains, which serve as epigenetic markers for the activation, repression, or poised states of gene transcription. Histone methyltransferases (HMTs) are divided into two families due to the function of methylate the lysine residues and arginine residues: histone lysine methyltransferases (KMTs) and protein arginine methyltransferases (PRMTs) [17]. However, studies of histone demethylase focus on histone lysine demethylation, and catalytic enzymes for arginine demethylation are rarely ­mentioned. What’s more, according to the different reaction mechanisms of demethylation, histone demethylase can be divided into two families: the Lysine-Specific Demethylases (LSD) demethylases and the Jumonji C domain-containing demethylases (JMJC) [18]. Histone methylation is dynamically changing during hematopoietic development and lineage specification, and histone methylation and related regulators play a crucial role in these processes, especially in erythropoiesis (Figure 1).

### 2.1. Histone lysine methylation

In mammalian cells, six histone lysine methylation sites have been fully studied: K4, K9, K27, K36, K79 and K20 on histone H3 or H4. Transcriptional effects are related to different methylation sites and degrees of methylation. The dimethylation and trimethylation of H3K4, H3K36 and H3K79 normally marks transcriptional activation, while H3K9, H3K27 and H4K20 methylation represent transcriptional inhibition [19,20].

#### 2.1.1. H3K4 methylation

Variations in the methylation levels of H3K4 are indicative of distinct biological functions. H3K4me1 is predominantly localized at enhancer regions and serves as a characteristic epigenetic marker of active enhancers. H3K4me3 usually occur in the gene promoter regions and is regarded as the activation signal of the gene transcription. In addition, differential H3K4 methylation states can also interact with other epigenetic modifications such as H3K9 acetylation and DNA methylation to participate in complex gene expression regulatory networks [21].

In mammals, H3K4 methylation is mainly modificated by methyltransferases of the histone-lysine N-methyltransferase 2 (KMT2) family, also known as mixed-lineage leukaemia (MLL) family. The family consists of six members: KMT2A (MLL1), KMT2B (MLL2), KMT2C (MLL3), KMT2D (MLL4), KMT2F (also known as SET Domain Containing 1 A, SETD1A) and KMT2G (also known as SET Domain Containing 1B, SETD1B). The methyltransferase activity of these MLL proteins are limited, especially MLL1 needs to form a protein complex with WD Repeat-containing Protein 5(WDR5), Retinoblastoma Binding Protein 5 (RBBP5), ASH2 Like, Histone Lysine Methyltransferase Complex Subunit (ASH2L), in order to effectively complete the methylation modification process [22].

MLL1 can participate in all methylation forms of H3K4, and its gene translocation rearrangement can lead to mixed lineage leukemia [22,23]. Several studies have shown that MLL1 affects the hematopoietic development system and *Mll1* knockout mice was embryonic lethal [24–26]. Although MLL1 has been shown to be essential for hematopoietic proliferation and lineage differentiation, this process does not seem to require the involvement of methylase activity [22,23,27]. The SET domain, and thus HMT activity of MLL1 is not necessary for hematopoietic target gene regulation [27].

MLL2 is highly similar to MLL1 but does not participate in chromosomal translocation [28]. MLL2 plays an important role in macrophages in the hematopoietic system and regulates the expression of β-globin during terminal erythroid differentiation [29,30]. MLL2 is recruited to the β-globin locus *via* interacts with Nuclear factor erythroid 2(NF-E2), thereby regulates the H3K4me3 levels of β-globin coding region. The knockdown of MLL2 in MEL cell decreased β-globin transcription and H3K4 trimethylation during erythroid differentiation [30].

The trimethylation of H3K4 is more efficiently catalyzed by SETD1A/B compared to MLL1. SETD1A promotes the expression of genes related to DNA damage and repair, and maintain cell proliferation and differentiation in HSCs [31]. *Setd1a*-CKO mice exhibited reduced levels of Ter119/CD71 positive erythroblasts, peripheral blood RBCs, and hemoglobin in the erythroid compartment. The absence of SETD1A resulted in reduced promoter-associated H3K4 methylation, suppression of gene transcription, and blockage of erythroid differentiation [32]. The impact of SETD1B deletion on hematopoietic systems is more severe than that of SETD1A. This is attributed to the crucial role SETD1B plays in maintaining the expression of key lineage-determining factors, including CCAAT enhancer binding protein alpha(Cebpa), GATA Binding Protein 1(Gata1*)*, KLF Transcription Factor 1(Klf1), mediated by H3K4me3 at gene promoters. Consequently, the deletion of Setd1b leads to fatal hematopoietic defects and compensatory hematopoietic proliferation in mice [33]. The expression of transcription factors of erythroid lineage differentiation such as Gata1 and Klf1 was down-regulated after Setd1b deletion. Phenotypically, *Setd1b*-CKO mice showed macrocytic anemia, which was characterized by a significant reduction in the number of red blood cells, lower hemoglobin concentration, higher mean corpuscular volume (MCV)and increased numbers of circulating reticulocytes [33].

H3K4 methylation modification is eliminated by members of the LSD family, among which LSD1 (Lysine Demethylase 1 A, KDM1A) is the most extensively investigated. LSD1 primarily functions at transcriptional initiation sites and enhancers. Apart from demethylating H3K4me1/2, it is also capable of demethylating H3K9me1/2 [34,35]. LSD1 is involved in γ-globin silencing during adult erythropoiesis. Treatment with an inhibitor of LSD1 upregulated γ-globin expression by increasing H3K4me2 levels in the γ-globin promoter region. Therefore, LSD1 is considered a promising therapeutic target for β-globinopathies, particularly sickle cell disease and β-thalassemia [36,37]. In addition, LSD1 is associated with erythroid-specific transcription factors including GATA-1, C/EBPa and TAL BHLH Transcription Factor 1(TAL1), which regulate hemoglobin synthesis and erythroid differentiation [36,38,39]. LSD1 plays a beneficial role in erythropoiesis by modulating HSCs differentiation potential, specifically promoting a shift from myeloid lineage commitment toward erythroid lineage development. In contrast, inhibition of LSD1 activity leads to the reactivation of PU.1-dependent enhancers, resulting in the disruption of erythroid transcriptional programs and concomitant expansion of myeloid progenitor populations, which are characterized by high proliferative and inflammatory potential [36,40,41].

#### 2.1.2. H3K9 methylation

H3K9 methylation is commonly considered a marker of heterochromatin due to its role in providing a binding site for heterochromatin protein factor (HP1), which can recruit additional methylases to amplify the effect [42]. In mammals, the maintenance of H3K9 methylation homeostasis is orchestrated by three major methyltransferase families: SUV39H1 histone lysine methyltransferase and SUV39H2 histone lysine methyltransferase(SUV39H1/2), SET domain bifurcated histone lysine methyltransferase 1/2(SETDB1/2), and the euchromatic histone lysine methyltransferase 2/1(EHMT2/1 or G9A/GLP) complex, which exhibit substantial functional redundancy while cooperatively regulating genomic integrity.

The level of H3K9me3 in HSCs isolated from both elderly humans and aged mice has been found to decrease. The Suv39h1/Suv39h2-mediated H3K9me3 has been implicated in the senescence of HSCs. This is supported by observations in Suv39h1/Suv39h2 double knockout mice, where HSCs exhibit reduced stem cell function along with characteristic markers of cellular aging. Specifically, these HSCs show an increased number of nucleoli and elevated levels of phosphorylated histone H2AX (γ-H2AX) foci, which is indicative of DNA damage accumulation [8,43]. However, overexpression of SUV39H1 induced erythroblasts immortalize and abnormal erythroblasts differentiation in *SUV39H1* transgenic mice [44].

SETDB1 is the only autosomal histone methyltransferase that catalyzes H3K9me3. The mechanism by which SETDB1 regulates hematopoietic lineage differentiation is complex. Wu et al. demonstrated that Setdb1 deficiency in zebrafish leads to impaired T cell development and erythropoiesis, accompanied by enhanced myeloid lineage differentiation, because of abolished H3K9me3 in hematopoietic regulatory genes such as CCAAT/enhancer-binding protein β (C/EBPβ) and Cyclin Dependent Kinase Inhibitor 1a (CDKN1A) [45], while additional research showed mice with Setdb1 deleted reduced H3K9me3 levels of non-hematopoietic lineage related genes such as fructose-1, 6-diphosphatase 1(*Fbp1*) and fructose-1, 6-diphosphatase 2(*Fbp2*), which was ectopic activation and resulting in antagonized glycolysis and impaired ATP production, farther leaded to the rapid depletion of hematopoietic stem/progenitor cells (HSPCs) [45,46]. Studies have shown that mice with Setdb1 deleted using the *Rosa:Cre^ERT^* method experienced rapid consumption of HSPCs except megakaryocyte/erythrocyte progenitor cells significantly decreased [46].

The establishment of de novo mono- and di-methylation at H3K9 exhibits high dynamics in the hematopoiesis, and the methylation is primarily mediated by G9A (EHMT2) and its highly homologous partner GLP (EHMT1), both members of the Su(var)3-9 family of methyltransferases. In primitive HSCs, the level of H3K9me2 is low, and the deletion of G9A did not impact HSC functionality. During the subsequent early differentiation process, H3K9me2 gradually spread to most gene regions in committed HSPCs. During erythropoiesis, G9A and GLP can regulate the silencing of *HBE1*, *HBG1* and *HBG2* genes and inhibit the synthesis of fetal hemoglobin (HbF) by altering the chromatin secondary structure of LCR and fetal hemoglobin genes. Eventually, H3K9me2 gradually disappeared and the deletion of H3K9me2 at this stage has not been shown to affect the expression of key transcription factors such as GATA1, KLF1 and NFE2 during terminal differentiation [47–49].

On the contrary, histone demethylase PHD Finger Protein 2(PHF2) is involved in the dynamic process of H3K9me2 in hematopoietic development through antagonism with histone methyltransferase (HMT). PHF2 expression progressively declines during the differentiation of K562 cells and human CD34^+^ hematopoietic progenitor cells toward megakaryocytic (MK) and erythroid lineages, but is subsequently restored during the terminal maturation phase of both lineages. Additionally, p53 as a negative regulatory factor involved in megakaryocytic and erythrocyte differentiation, exhibited expression patterns similar to those of PHF2. Research by Jichun Yang et al. has demonstrated that PHF2 can regulate p53 expression by removing H3K9me2 on its promoter region. This highlights the significant role played by H3K9me2 in influencing MK and erythroid differentiation [50].

#### 2.1.3. H3K27 methylation

Similar to H3K9 methylation, histone H3K27 methylation is also an inhibitory modification necessary for heterochromatin formation. The distribution of different forms (H3K27me1/2/3) throughout the genome is mutually exclusive. H3K27me3 mainly located in the promoter region and leads to compactness of chromatin structure. H3K27me2 primarily found in intergenic regions, while H3K27me1 is predominantly present in the body of genes with transcriptional activity [51].

The key enzymes involved in the modification of H3K27me1/2/3 are Enhancer Of Zeste Homolog 1/2 (EZH1/2), which typically assemble into Polycomb Repressive Complex 2 (PRC2) alongside Embryonic Ectoderm Development(EED), Suppressor Of Zeste 12(SUZ12), Retinoblastoma-binding protein 46/48 (RbAp46/48), and other cofactors to execute their respective biological functions. They are believed to play a crucial role in the maintenance of HSCs [52–54]. However, due to functional redundancy, the deletion of these two enzymes alone did not have a significant impact on adult HSCs [55,56], although early research shows that EZH2 is also essential for fetal HSCs [54,56,57]. During the erythropoiesis, H3K27me3 is exported from the nucleus to the cytoplasm [58]. EZH2, which is always located in the nucleus, regulates the differentiation and growth of erythroid progenitor cells in a H3K27me3-dependent manner in the early stage of erythropoiesis, and maintains the enucleation of terminal erythrocytes independent of H3K27me3 but methylated the non-histone protein HSP70 in terminal erythroid differentiation [58]. Furthermore, some studies have indicated that EZH2’s maintenance of normal hematopoietic function in zebrafish is independent of its histone methylase activity [59]. However, pharmacological inhibition of EZH2 using GSK126 in erythroid cells induces the Bcl-2 interacting mediator of cell death(Bim) expression with global reduction of H3K27me3 levels, resulting erythroid cells apoptosis, EZH2 activity is required for protecting human erythroblasts [60].

The erasure of H3K27me3/2 modifications is primarily mediated by the KDM6 family including Lysine Demethylase 6 A(KDM6A, also known as UTX), Lysine Demethylase 6B(KDM6B, also known as JMJD3), and Lysine Demethylase 6 A (KDM6C, also known as UTY). Interestingly, UTX can maintain hematopoietic homeostasis with or without demethylase activity by removing deposition of H3K27me3 at transcriptional initiation sites and protecting the function of HSCs [61]. Unlike UTX which is stably expressed in most tissues, JMJD3 expression is induced under various stress or pathogenic factors and has been associated with malignant hematopoiesis [62]. In comparison to UTX, UTY on Y chromosome has lower catalytic activity but shares high similarity with UTX and can partially compensate for its loss in male individuals [63].

#### 2.1.4. H3K36 methylation

Histone H3K36 methylation serves as one of the markers of classical active chromatin and is closely associated with intragenic methylation. Currently, there are at least eight kinds of HMT enzymes capable of catalyzing H3K36 methylation, included SET Domain-Containing Protein 2(SETD2 or KMT3A), Nuclear Receptor Binding SET Domain Protein 1(NSD1 or KMT3B), Nuclear Receptor Binding SET Domain Protein 2(NSD2 or KMT3G), Nuclear Receptor Binding SET Domain Protein 3(NSD3 or KMT3F), Absent, small, or homeotic1-like(ASH1L), SET Domain and Mariner Transposase Fusion Gene (SETMAR), SET and MYND Domain Containing 2(SMYD2) and SET Domain Containing 3(SETD3).

SETD2 is the only known mammalian methyltransferase capable of catalyzing H3K36me3 independently of presence of H3K36me1 and H3K36me2. Studies have shown that *Setd2*-null mouse embryos were lethal, exhibited severe anemia [64,65]. During hematopoiesis, Setd2 is widely expressed in many hematopoietic subsets, with relatively high expression in HSPCs, but low expression in mature myeloid and erythroid cells. Setd2 contributes to the self-renewal capacity of HSCs to some extent. HSCs deleted Setd2 with H3K36me3-deficient exhibit loss of quiescence, increased apoptosis and decreased pluripotent differentiation potential [66]. Interestingly, studies have shown that the mice deleted Setd2 leads to abnormal hematopoiesis resulting in depletion of phenotypic and functional HSCs with a significant decrease in the number of multilineage progenitor cells, there is a sharp increase in erythroid lineage such as erythroid progenitors and primordial erythrocytes due to the down-regulation of H3K36me3, which enhanced the RNA polymerase II elongation while activating expression of erythroid lineage regulators such as *Gata1*, *Gata3*, *Zfpm1*, *Klf1* and *Gfi1b* [64,67]. Other studies have shown that loss of Setd2 delay the differentiation and decrease enucleation during the terminal erythropoiesis. Therefore, Setd2 deficient mice were severely anemic despite the number of erythroid progenitor cells increased significantly [65]. Similarly, NSD1, which catalyzes H3K36me1 and H3K36me2, activity regulated chromatin binding and target gene activation by the key transcription factors GATA1. The deletion of NSD1, which catalyzes H3K36me1 and H3K36me2, profoundly inhibits the maturation of terminal erythrocytes, and there is a tendency to accumulate and transform malignant erythroleukemia [68].

H3K36 methylation is also reversible which removed by Jmjc demethylases include the KDM2/JHDM1 and KDM4/JHDM3/JMJD2 family proteins [69,70]. KDM2B is a Jumonji (JmjC) domain histone H3K36 di-demethylase (H3K36me2). Jaclyn et al. found that KDM2B is essential for maintenance and lineage commitment of HSPCs, *Kdm2b*-null mice exhibited increased myeloid differentiation and decreased lymphoid differentiation, although the number of RBCs had no change in peripheral blood [71].

#### 2.1.5. H3K79 methylation

In hematopoietic cells, H3K79me2/3 modification is associated with active transcription of chromatin regions, while genes modified only by H3K79me1 typically struggle to maintain a high level of expression [72]. Disruptor of telomeric silencing 1-like (DOT1L), a homologue of DOT1, is the only HMT in mammals capable of catalyzing H3K79me1/2/3 [73]. There is no demethylase that specifically removes H3K79 methylation, and several Jmjc family proteins regulate H3K79 methylation, include KDM2B, KDM7A/B, which also removes other lysine methylations [74,75]. Compared with other HMTs, DOT1L has some unique structural and biochemical characteristics. Firstly, DOT1L lacks the characteristic SET domain and utilizes a special region of its C-terminal to catalyze the methyl transfer reaction. Additionally, the substrate residue H3K79 on DOT1L is located within a ring on the surface of nucleosome’s histone globular domain, rather than in the N-terminal tail of the H3 protein [76].

DOT1L has been proved to play an important role in embryonic development as embryos with DOT1L deletion exhibit severe anemia and perish before E13.5 [77]. In order to investigate the role of HMT activity of DOT1L in this process, *DOT1L*-mimic (*DOT1L-MM*) embryonic mouse model without methyltransferase activity was constructed [77,78]. Although lethality was observed in this model as well, it did not display any related hematopoietic defects [77,78]. This suggests that H3K79 methylation modification appears unnecessary for primitive erythropoiesis.

Considering the high H3K79 methylation status during hematopoietic differentiation, previous studies have suggested that H3K79 methylation is involved in the myeloid/erythroid fate transition of progenitor cells. This is supported by enrichment of histone modification found on the regulatory elements of GATA2 and PU.1 [76]. However, recent gene expression analysis has shown that there is no corresponding change in the expression of *GATA2* and *GATA1* in mice treated with EPZ-5676 (an inhibitor of DOT1L) [77]. Furthermore, during the culture of *DOT1L-MM* mouse model *in vitro*, the development of yolk sac cells and the capability of erythroid-lineage progenitors to form colonies were essentially normal [77]. This evidence suggests that loss of H3K79 methylation does not block the erythropoiesis. However, in contrast to erythroid colonies, myeloid and mixed colonies of *DOT1L-MM* mice decreased significantly [77]. In fact, H3K79 methylation levels have been found to show dynamic changes from high to low during myeloid development [72]. Therefore, these data suggest that H3K79 methylation significantly affects the development of myeloid progenitor cells rather than erythroid progenitor cells.

In addition, both DOT1L-KO and DOT1L-MM cells showed a certain degree of DNA damage characterized by the accumulation of G0/G1 cells and an increase in apoptosis [76]. The mechanism behind this evidence is that the deletion of H3K79 methylation not only leads to down-regulation of *HOX* gene essential for the self-renewal of HSCs [72], but also results in a significant increase of Cyclin Dependent Kinase Inhibitor 1 C (*CDKN1C*) [78]. This indicates that H3K79 methylation is involved in both gene activation and gene inhibition in the process of hematopoiesis. Although this effect may be indirect, H3K79 methylation affects the expression of genes related to hematopoietic progenitor cells (HPCs) development and renewal in late embryonic hematopoiesis. DOT1L is also involved in the hematopoietic process of adults. The *Dot1l* conditioned knockout mice showed a phenotypic decrease in the number of bone marrow small cells and HSPCs chambers, therefore the Dot1l deletion resulted in severe anemia in internal organs such as the liver [79,80].

#### 2.1.6. H4K20 methylation

H4K20 methylation modification plays a crucial role in various significant biological processes, such as chromatin compression, DNA damage repair [81,82]. The mono-methylation modification at this site is involved in the regulation of transcription inhibition and activation. Similarly, di-methylation modification is similar to mono-methylation modification but with different distribution patterns, while trimethylation modification is associated with heterochromatin formation [81,83].

In mammals, SET Domain Containing 8(SETD8) serves as the sole histone methyltransferase capable of catalyzing H4K20me1, and SETD8 is significantly higher expression during erythrocyte maturation compared to other cell types or tissues [82,84,85]. Research suggests that SETD8 can induce the transformation of proerythroblasts into terminal erythroid differentiation stage [86]. In contrast, knockdown of SETD8 showed phenotypes such as low enucleation rate and delayed hemoglobin accumulation indicating block the terminal erythroid differentiation [86,87]. In addition, embryos with Setd8 deletion almost completely lost H4K20me1 and died from severe anemia at E11.5 due to increased erythrocyte apoptosis [88].

Transmission electron microscope (TEM) and ATAC-seq analysis revealed defects in chromatin condensation and heterochromatin accumulation in Setd8 ^Δ/Δ^ erythrocytes [84,88], suggesting that functions of SETD8 primarily regulated the erythrocyte maturation as an inhibitory factor. It can be inferred that SETD8 acts as a co-inhibitor of GATA1 by participating in the transcriptional inhibition of GATA2. Andrew W. et al. propose that the mechanism involved SETD8-catalyzed H4K20me1 limiting the occupation of GATA2 transcriptional enhancer Scl/TAL1 [86]. However, the effect of knock down the GATA-2 on erythrocyte maturation varies across different studies. These discrepancies may indicate that SETD8 regulation of erythrocyte maturation involves multiple target genes including *HES1*, *HHEX*. The up-regulation of these genes were observed following SETD8 depletion [87,88].

Following SETD8-mediated H4K20me1 catalysis, Suppressor of variegation 4–20 homolog 1/2 (SUV420H1/2) can recognize and catalyze di-methylation and tri-methylation at this site, promoting further chromatin condensation [89]. Amuri196, as an inhibitor of SUV420H1/2, minimally interfered with the proliferation and differentiation of K562 cells [90]. Additionally, SUV420H1 has been implicated in silencing the ε-globin gene in primary erythroid progenitor cells [91]. Furthermore, transcriptome analysis revealed up-regulation in *SUV420H2* gene expression under the stimulation of erythropoietin(EPO), suggested that a potential role for SUV420H2 in erythropoiesis [92]. These findings suggest that H4K20 methylation modification play a significant role in extensive transcriptional inhibition during erythropoiesis.

### 2.2. Histone arginine methylation

Histone arginine methylation is carried out by the protein arginine methyltransferases (PRMT) family consisting of nine members (PRMT1-9), which can be categorized into three types based on their form of methylation: type I (PRMT1-4,6 and 8) catalyzes asymmetric dimethylarginine formation; type II (PRMT5 and PRMT9) catalyzes symmetrical dimethylarginine formation, and type III (PRMT7) is believed to solely facilitate monomethyl arginine (MMA) formation.

#### 2.2.1. H3R2 methylation

H3R2me2a can be catalyzed by type I arginine methyltransferase PRMT6 and plays a crucial role in megakaryocyte/erythroid branches. During megakaryocyte differentiation, PRMT6-mediated H3R2me2a modification inhibits the expression of various erythroid-specific genes such as *AHSP*, *ALAS2*, *GYPA* and *KLF1*. Notably, the disruption of the interaction between WD Repeat-Containing Protein 5 (WDR5), a core component of the MLL complex, and protein arginine deaminase PADI4 at the GYPA promoter leads to a decrease in active H3K4me3 modification and establishes an inhibitory chromatin environment characterized by H3K27me3 through H3R2me2a labeling. Conversely, down-regulation of H3R2me2a by MS023 relieves inhibition of erythroid-specific genes, promotes terminal erythroid differentiation [93]. Previous studies have also indicated that PRMT6 can interact with other epigenetic regulators such as members of the polycomb complex (PRC) and regulate cell proliferation and senescence [94–97].

It is noteworthy that deletion of type I arginine methyltransferase PRMT4 or type III arginine methyltransferase PRMT7 has minimal effect on hematopoietic function. The former specifically catalyzes asymmetric di-methylation of H3R17 and H3R26, which can be compensated by other type I arginine methyltransferases upon deletion. The latter mediates arginine mono-methylation of histone 4(MMA), but there is no evidence supporting its role in erythropoiesis [98,99].

#### 2.2.2. H4R3 methylation

PRMT5 is the main type II PRMT enzyme, forming a heteroatomic complex with alpha matrix protein 50(MEP50) to mediates the symmetrical di-methylation of H4R3 and H3R2, H3R8 and H2AR3 that typically inhibit gene transcription [100]. Among these modifications, it has been reported that H4R3me2s inhibits expression of cell cycle regulatory genes including *cyclinE1*, *RB*, *P14ARF* and *P16INK4a* [101–103]. Studies have shown that absence of Prmt5 in mice almost completely eliminates symmetrically di-methylated arginine modification leading to fatal pancytopenia, especially loss of Prmt5 impairs erythroid differentiation with an reduced CD71/Ter119 double-positive cells in the BM and spleen [104].

There is also asymmetric di-methylation(H4R3me2a) at the H4R3 site, which is catalyzed by PRMT1. H4R3 methylation is necessity for transcriptional activation. In erythroid differentiation, inhibition of PRMT1 depleted H4R3me and increased H3K9me and H3K27me on the β-globin locus [105]. Studies have shown that the absence of Prmt1 in mice can led to reduced terminal erythroid differentiation and anemia. Moreover, the results of BM transplantation showed that Prmt1-null led to a decrease in the self-renewal ability of HSPCs [106].

## 3. Acetylation modification of histones

Acetylation was identified as one of the earliest histone modifications affecting transcriptional regulation and is therefore currently the most studied. Increased histone acetylation abundance has long been thought to be associated with reduced chromatin and active gene expression. Acetylation regulates important cellular processes, including normal and abnormal erythropoiesis by altering chromatin epigenetic state [107]. Different histone acetyltransferases (HATs) and histone deacetylases(HDACs) sequentially provide fine regulation for erythroid progenitor development by controlling gene expression or regulating non-histone function [108]. In particular lysine acetylation in histone tails is a highly dynamic and important process that plays a crucial role in the regulation of chromatin structure, transcription and DNA repair. In humans, three major families of HATs have been well studied, including Gcn5-related N-acetyltransferase(GNAT includes HAT1, GCN5, PCAF), MOZ, Ybf2/Sas3, Sas2, Tip60 (MYST includes Tip60, MOF, MOZ, MORF, HBO1), and E1A-binding protein p300/CREB-binding protein (p300/CBP). These HATs primarily modify specific sites on histones such as K9, K14, K23, and K27 of histone 3 and K5 and K9 of histone 4 to maintain the hematopoietic homeostasis [109,110]. On the other hand, HDACs can be divided into four categories based on their sequence and tertiary structural homology with yeast HDACs. The first category includes RPD3 homolog: HDAC1, 2, 3, and 8. The second class is related to yeast HDA1 protein and can consists of two subsets: class IIa(HDAC4, 5 and 7) and class IIb(HDAC6 and 10). The third class comprises sirtuins 1–7, whose catalytic activity depends on the coenzyme NAD^+^. Lastly, the fourth class only contains HDAC11 [111–113]. These enzymes play a critical role in covalently modifying lysine residues on core histones at specific sites such as K9, K27 and K56 of histone 3, affecting not only gene expression but also non-histones regulation.

**Figure 2.**
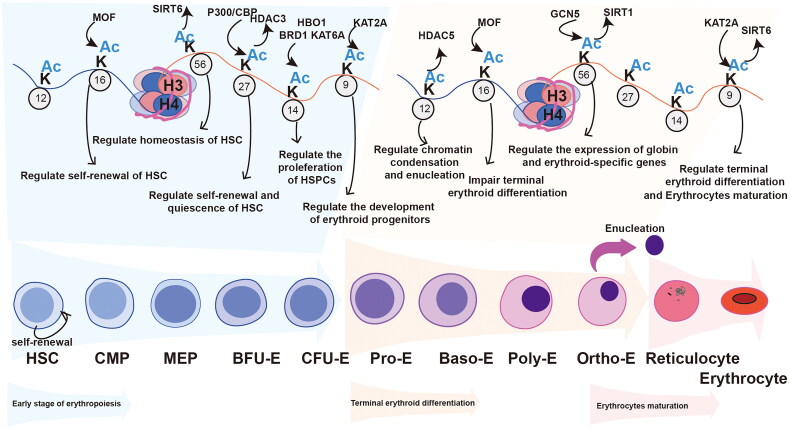
The role of histone acetylation in erythropoiesis. Erythropoiesis is the process of HSC differentiation into erythrocyte, which is mainly divided into three stages: the early erythropoiesis stage (light blue differentiation path), terminal erythroid differentiation stage (light orange differentiation path), and erythrocyte maturation stage (pink path). The left part of the diagram shows the role of histone acetylation and related modifying enzymes (numbers representing methylation sites, K representing lysine residues, protein-directed acetylation indicating that the protein is acetyltransferases, and acetylation-directed protein indicating that the protein is deacetylases) in early erythropoiesis. It is mainly involved in regulating the function of hematopoietic stem progenitor cells and the development of erythroid progenitor cells. The right part of the diagram shows the role of histone acetylation and related modification enzymes in the terminal erythroid differentiation, which mainly regulates chromatin condensation, enucleation and globin expression in erythroblast.

Next, we mainly reviewed the role of lactated modification of related histones in hematopoietic development, especially in erythropoiesis (Figure 2).

### 3.1. H3K9 acetylation

Genome-wide studies have shown that H3K9ac is part of the active promoter state present on bivalent promoters and active enhancers along with other ‘active’ histone modifications (e.g. H3K14ac, H3K27ac, H3K4me3 and H3K27me3) to establish transcriptionally compatible chromatin conformations for regulating downstream gene expression [114]. In addition, recent studies have revealed that the loss of H3K9ac modification had significant impact on acetylation at nearly all H3 and H4 sites. This suggests that the presence of H3K9ac is essential for subsequent acetylation establishment at other sites [115].

During erythropoiesis, acetylation of H3K9 is mainly mediated by the Lysine Acetyltransferase 2 A(KAT2A) of the GCN5 family. As part of the HAT module, KAT2A is frequently involved in forming two different macromolecular complexes, Ada-two–A-containing(ATAC) and Spt-Ada-Gcn5 acetyltransferase(SAGA) [116]. Knockdown of the HAT module activity of ATAC and SAGA in mammalian cells significantly reduced H3K9ac levels at all active gene promoters. These two macromolecular complexes play a stage-specific role in erythroid progenitor development. The ATAC complexes can regulate erythroid progenitor specification and survival. While the SAGA complex is thought to have a possible role in terminal erythroid differentiation [117,118]. Studies have shown that impairment of the ATAC complex leads to specific defects in erythroid specification in HSCs, while reducing H3K9 acetylation levels at the Erythropoietin receptor(EPOR) promoter, affecting EPOR expression to some extent [118].

Deacetylation of H3K9 is also important epigenetic mechanism during erythrocytes maturation. Acetylation of H3K9 is associated with active gene transcription, whereas deacetylation of H3K9 is associated with gene repression [119]. Sirtuin 6(SIRT6) serves as a specific histone deacetylase for H3K9ac. Hyperacetylation could be observed at SIRT6-deficient cells specifically for other histone residues, consistent with the specificity for this particular modification. Previous studies have found that SIRT6 can regulate the chromatin structure of telomeres, thereby impacting the aging of HPCs. Mechanistically, SIRT6 is recruited to chromatin at RELA Proto-Oncogene, NF-KB Subunit(RELA) target gene promoters through physical interactions with the subunit RELA, leading to deacetylating of histone H3K9 [120]. This deacetylation results in RELA instability and subsequent chromatin modification of target genes, ultimately terminating NF-κB signaling. Given that NF-κB signaling is involved in the expression of genes associated with cellular senescence in HPCs [121], the precise regulation of NF-κB activity by SIRT6 plays an important role in the maturation and aging of erythrocytes.

### 3.2. H3K14 acetylation

Lysine acetyltransferase 7 (KAT7, also known as HBO1), a member of the MYST acetyltransferase family(also known as KAT7 or MYST2), can form various HAT complexes with different scaffold proteins and act on multiple histone acetylation sites. The recently study identified Hbo1-Brd1-HAT complex is responsible for regulating H3K14 acetylation during fetal liver erythropoiesis [122]. Knockdown of Hbo1 or deletion of Brd1 leads to a significant reduction in intracellular H3K14 acetylation levels [122]. Additionally, Lysine Acetyltransferase 6 A(KAT6A, also known as MOZ), another member of MYST acetyltransferase family, has been shown to regulate H3K14ac as well [123], and its HAT activity is essential for the proliferation of early hematopoietic precursors [124].

Both HBO1 and MOZ play important roles in maintaining the function of HSCs [122,123, 125,126]. Deletion of both enzymes separately resulted in mid-gestation embryos death and impaired fetal liver erythropoiesis [122,123]. Decreased H3K14ac levels resulting from Hbo1 or Brd1 depletion not only interfered with erythroid progenitor differentiation, but also resulted in massive apoptosis of erythroblasts in fetal liver [122]. Whereas loss of MOZ neither prevented final differentiation of mature erythrocytes nor affected levels of H3K14ac in erythroblasts [123,127]. Abnormal Gata1 expression as one of the causes of *Brd1^-/-^* embryonic anemia [122]. In addition, the deletion of H3K14ac can affect a variety of hematopoiesis-related genes, including *Mpl*, *Gata2*, *Meis1* and *Hox* [126]. The downregulation of these genes severely disrupts the balance of self-renewal and proliferation ability of HSCs, leading to a decrease in the proportion of HSCs in G0 phase and a loss of HSC numbers [126].

### 3.3. H3K27 acetylation

There is antagonism between H3K27ac and H3K27me3 during to share a locus. Depletion of PRC2 promotes a global increase in H3K27 acetylation levels catalyzed by P300 and CBP [107]. CBP primarily functions in HSC self-renewal and selectively increases myeloid differentiation following deletion, while P300 is required for hematopoietic differentiation [128,129]. Surprisingly, as a multidomain transcriptional coactivator, the enzymatic activity of P300’s HAT domain is far less important than KIX and CH1 domains in hematopoiesis [130], suggesting that P300-catalyzed H3K27ac may not provide critical functions in hematopoietic development.

Histone deacetylase 3(HDAC3), a Class I HDAC family member, shares homology with the yeast deacetylase RPD3 and is responsible for the comprehensive deacetylation modification of the histone H3K27 residue. HDAC3 is considered a major epigenomic modulator in the nucleus that helps regulate gene expression, chromatin structure and genomic stability [131]. HDAC3 is indispensable for HSPCs functionality during the S phase, as well as for stem cells and lymphopoiesis, as evidenced by experiments involving conditional deletion alleles in mice and DNA fiber labeling assays [132]. Moreover, the oncogene DEK recruits HDAC3 to induce deacetylation of H3K27, thereby limiting chromatin accessibility in HSCs and transcriptionally regulating the expression of genes associated with HSC quiescence(e.g. *Akt1/2*, *Ccnb2*, and *p21*) [133,134].

### 3.4. H3K56 acetylation

Acetylation modifications at H3K56 are distinct from other sites and play a key role in genome-wide activators of transcription [135]. The dynamic balance between acetylation and deacetylation of H3K56 is essential for genomic stability maintenance. In mammals, P300 and GCN5 engagement accomplish acetylation of H3K56 while SIRT1 and SIRT6 mediated its deacetylation [136]. GCN5 regulates the expression globin and erythroid-specific genes *via* interact with ZBP-89 to acetyl-ate H3 [137]. As NAD-reliant histone deacetylases, sirtuin family proteins effectively remove acetyl groups from histones, thereby preventing transcription factors from accessing DNA and safeguarding HSCs from the effects of aging. Among them, SIRT1 mainly plays an important role in myeloid and lymphoid specification [138]. Other studies have shown that SIRT1 does not affect the proliferation and differentiation of erythroid progenitors, but can activate the expression of γ-globin [139]. SIRT6 plays an important role in HSC homeostasis. Sirt6 deletion in adult mice results in aberrant activation of Wnt signaling, which leads to aberrant HSC proliferation and subsequent functional defects [140,141].

### 3.5. H4K12 acetylation

H4K12 acetylation was significantly reduced at late stages of erythroblast differentiation, and histone deacetylation was directly responsible for chromatin condensation. It is worth mentioning that six of the eleven classical members of the HDAC family(HDAC1-3 and HDAC5-7) are expressed in human erythroid cells [12]. Whereas during terminal differentiation, except HDAC5, which increases significantly, the remaining HDAC members either decrease significantly (HDAC1-3) or change little(HDAC6 and HDAC8) [12,113, 142,143]. Analyses using ATAC-seq and RNA sequencing demonstrated that knocking down HDAC5 results in enhanced genome-wide chromatin accessibility and widespread alterations in gene expression. Studies have demonstrated that HDAC5 selectively accomplishes the deacetylation of H4K12 and exhibits notable expression at the terminal erythroid differentiation, which has a profound impact on chromatin condensation and enucleation [12].

In addition, the role of HDAC1 in the development of erythrocytes is independent of histone H3 and H4 deacetylation at promoters, and its target protein is actually a PU.1 Non-histone proteins associated with the core promoter region [144]. Other HDACs are mostly associated with malignancy and there is no clear evidence of acting on histones in normal hematopoiesis [145–147]. HDAC11, as the only member of class IV HDACs, is closely related to lineage determination of myeloid cells, differentiation and maturation of monocytes and granulocytes, but is not essential for differentiation of HSCs and HSPCs [148].

*In vivo* and *in vitro* experiments have shown that histone acetylation plays an essential role in the regulation of hemoglobin expression. The histone deacetylase inhibitor butyric acid and its derivatives have been identified as inducers of silenced embryonic and fetal β-type globin gene expression in several animal models [149,150]. Therapy of patients with sickle cell anemia and β-thalassemia with sodium butyrate and butyric acid induces increased HbF expression [151,152]. Increased fetal hemoglobin expression is associated with increased histone acetylation in the vicinity of the γ-globin gene [153]. Class I HDACs are able to interact with specific erythroid lineage transcription factors and thus play a role in the regulation of human γ-globin genes. For example, HDAC1 interacts with Ikaros and NF-E4 to regulate γ-globin gene expression [154,155]. HDAC3 binds to NCOR1(nuclear receptor co-repressor protein 1), a protein complex that regulates fetal globin gene expression by occupying/exiting the γ-globin gene promoter, which correlates with histone H3 and H4 acetylation at the γ-globin gene promoter. Furthermore, in human erythroid progenitor cells, specific siRNA knockdown of endogenous HDAC3 results in increased transcription of the γ-globin gene promoter [156]. In contrast to HDAC1 and HDAC3, HDAC9 binds to the promoter region of the γ-globin gene and upregulates its gene expression. Furthermore, knockdown of HDAC9 by siRNA in primary human erythroid progenitor cells resulted in silencing of the γ-globin gene. In contrast, forced expression of HDAC9 increased both γ-globin and HbF mRNA levels. In addition, multiple transcription factor binding regions for myocyte enhancer factor 2(MEF2) were found in γ-globin promoters, so MEF2 may be involved in the recruitment of HDAC9 to these promoters [157].

### 3.6. H4K16 acetylation

Chromatin regions labeled with H4K16ac showed higher accessibility. In mammals, it is the Lysine Acetyltransferase 8 (KAT8, also known as MOF) of the MYST family that completes the H4K16ac modification. Homozygous *Mof***-**null mice(*Vav1-cre*; *Mof^f/f^*) developed severe hematopoietic cytopenias, particularly HSPCs, and died about 10 days after birth. Mice with heterozygous *Mof* deletion also developed an anemic phenotype, however, they survived normally [158]. In this process, loss of H4K16ac has been shown to be a major contributor to anemia, as the HAT domain mutant Mof, did not rescue hematopoietic defects in *Mof*-null individuals experimentally [158].

This hematopoietic stem cell with low H4K16ac is considered to be an intermediate state of dHSC (dynamic hematopoietic stem cells) and aHSC (static hematopoietic stem cells), this cell upregulates some genes related to cell cycle and metabolism and has a stronger proliferative advantage. But increases in reactive oxygen species (ROS) and phosphorylated AKT-1(p-AKT-1) during serial transplantation show this proliferative advantage at the expense of impaired self-renewal capacity [159,160]. Furthermore, prior research has uncovered a decline in H4K16ac levels in tandem with the aging process of HSCs [161]. These findings suggest that H4K16ac contributes to the maintenance of HSC self-renewal capacity.

Unlike the balance between self-renewal and differentiation, which predominantly relies on HSCs during adult hematopoiesis, the process of hematopoiesis in early pregnancy is primarily dependent on the swift proliferative capabilities of HSCs. In this case, the *vav1-cre* conditional knockout *Mof* mouse model constructed by Daria G. Valerio scholars did not show minor effects on hematopoiesis until E17.5 in late pregnancy, which emphasized that H4K16ac is necessary to maintain hematopoiesis in adults rather than hematopoiesis in early pregnancy [158].

In addition, H4K16ac and MOF are phased expressed during hematopoiesis [160]. *Mof*± HSC colonies initially exhibited low levels of H4K16ac and aberrant increases in expression after 5 days. At this stage, erythroid progenitors and MEPs were significantly reduced in *Mof*± HSCs recipient mice, accompanied by myeloid tilt. After experiencing low expression at the HPPs and pMEP stages, H4K16ac and MOF would also be highly expressed at the MEP stage. Although deletion of MOF does not affect erythroid colony formation when the MEP stage progresses to lineage shaping, it does affect erythrocytes at the terminal differentiation stage, as evidenced by accumulation of morphologically defective erythrocytes and increases in reticulocytes [160]. The above data indicate that H4K16ac impacts early erythroid development and terminal differentiation in a phased manner. Mechanistically, Cecilia scholars believe that H4K16ac can regulate the regulatory network involved in Runx1 and Gfi1b and establish lineage specificity during hematopoiesis [160].

## 4. Other histone modifications

Other histone modifications such as ubiquitination, phosphorylation and succinylation also play important roles in erythropoiesis. As early as the 1980s, studies have shown that histone H2A ubiquitination increases in erythroid differentiation, and ubiquitination of H2A may have a role in the chromatin structure changes necessary for erythroid cell differentiation [162,163]. Ubiquitin-ligase polycomb repressor complex 1 (PRC1) and H2A-deubiquitinating enzymes (H2A-DUBs) regulate ubiquitination and deubiquitination of H2A-K119, and H2A-K119u is associated with transcriptional silencing. The Myb-like SWIRM and MPN domains containing protein 1(MYSM1) as H2A-DUB, which is necessary for erythroid differentiation. The increased oxidative stress and genomic instability of HSCs in MYSm1-deficient mice lead to the impaired of HSCs function and finally show a significant reduction in the number of red blood cells [164].

Regulatory erythroid kinase(REDK) can phosphorylate H2b and H3 and its expression correlates with erythroid differentiation. REDK deleted *via* treatment of human bone marrow with REDK antisense oligonucleotides increased the number of BFU-Es and CFU-Es colonies [165]. The phosphorylation of H2A.X at S139 and Y142 is required for nuclear condensation during terminal erythroid differentiation [166]. Loss of H2A.X or the Y142 kinase, bromodomain adjacent to zinc finger domain 1B(BAZ1B) impaired Caspase-Initiated chromatin condensation in erythroid cells. Another histone phosphorylation modification is H2b pS14, which is associated with apoptotic chromatin condensation, and is present in erithroid progenitor maturation [166].

Lysine succinylation (Ksu) has recently emerged as a post-transcriptional modification of histone [167]. There is a conserved succinylation site on H3, H3K79Ksu, which is catalyzed by KAT2A and marks the activation of gene transcription [168]. The levels of H3K79Ksu is significantly increased, while the expression of KAT2A is stabilized during erythroid differentiation. Knockdown of KAT2A leads to a decrease in H3K79Ksu on genes involved in the nuclear envelope and chromatin remodeling pathway including *XPO7*, *FOXO3*, and *HDAC6*, suggesting that H3K79Ksu may participate in chromatin condensation, enucleation, and reticulocyte maturation processes in erythropoiesis [169].

## 5. The cross-talk of histone modification

Histone modifications are dynamically changed and mutual interfered in erythropoiesis, various histone modifications synergistically or antagonistically regulate the expression of erythroid-specific genes. The levels of acetylation of H3 or H4 and H3K4me2/3 in *EPOR* gene locus were increased significantly, while the levels of H3K9me3 and H3K27me3 were gradually decreased [170]. H3K9me2 and H3K27me2 synergistically regulate the switch of γ-globin and β-globin expression in adult erythropoiesis. The inactivation of G9A significantly reduced the levels of H3K9me2 and H3K27me2 in γ-globin gene promoters to activate γ-globin expression with an increased of transcriptional activation marker H3K36me3, oppositely the expression adult β-globin was inhibited [49,171]. Histone modification related transcriptional activation or repression is associated with transcription factor binding genes loci. Erythroid-specific genes locus bound by GATA1, a key transcription factor for erythroid differentiation, was significantly correlated with the levels of H4K16Ac on genes loci and the levels of H3K27Ac and H3K4me1 on enhancers, while the lower expression of other hematopoietic lineage target genes such as *GATA2* and *c-KIT* during erythroid differentiation was correlated with the high levels of H3K27me3 [172]. The high expression of HDAC5 in the late stage of erythroid terminal differentiation regulates the reduction of H4K12 acetylation and increases the level of H3K9 methylation to promote chromatin condensation and enucleation of erythroblast [173]. PRMT6-catalyzed H3R2me2a often marks the establishment of an inhibitory chromatin environment characterized by H3K27me3 with a decrease in active H3K4me3 modification in various erythroid-specific genes [92]. H4R3me as a transcriptional activation marker has antagonistic effects with H3K9me and H3K27me in β-globin locus [104]. Overall, histone modifications are complex, showing different modification states in specific loci and specific differentiation schedules during erythropoiesis, definitely transcriptional activation modifications have synergistic effects while antagonistic transcriptional inhibition modifications are involved in the regulation of erythroid characteristic gene expression.

## 6. Conclusion

**Figure 3.**
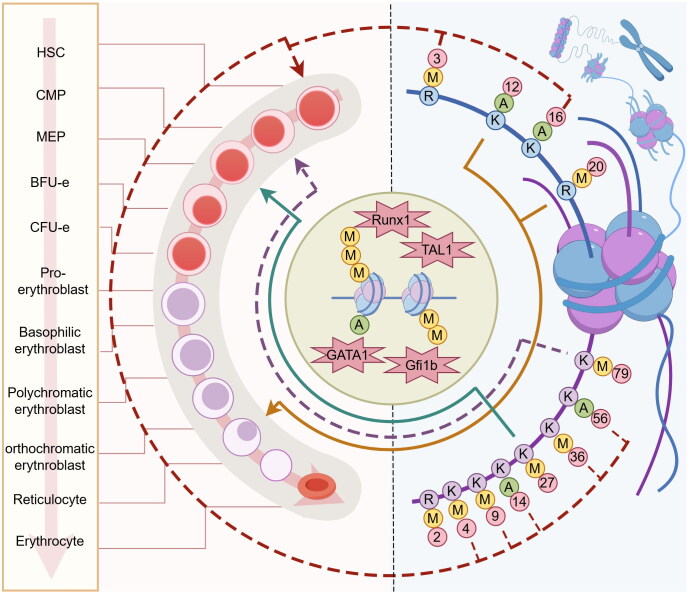
Schematic diagram of histone modification sites acting in erythropoiesis. The left side of the diagram shows the different cell lines during erythropoiesis including HSC, CFU-E, polychromatic erythroblast, reticulocyte, and so on. The right side of the diagram shows the main modification sites of H3 and H4 histones, and the information of the modification sites is marked on the N-terminal of H3 and H4 histones. The numbers show the position of each retouching. The letters indicate the amino acid or type of modification at each modification site: K representing lysine residues, R representing arginine residues, M representing methylated modification, and A representing acetylated modification. In addition, the diagram also shows that different modification sites and types are involved in the differentiation process of different stages of erythropoiesis by altering the expression of key genes such as Runx1, GATA1, etc.

We review here the modifications of histones, mainly the role of methylation and acetylation of histones in erythroid development (Figure 3, Tables 1 and 2). This epigenetic form impacts all DNA-based processes, including chromatin compaction, nucleosome dynamics and transcription. In the hematopoietic stem cell stage, modifications such as H3K9me3, H3K36me3, H3K27ac and H4K16ac help to maintain homeostasis in HSCs [8,66, 134,159]. Modifications including H3K4me3, H3K36me3, H3K14ac can affect the expression of the key lineage determinant GATA1 and regulate further differentiation of erythroid progenitor cells [33,67,122]. At the final erythroid maturation stage, H4K20me1 and H3K14ac played an important role in the condensation of chromatin [12,84]. Given that a range of critical histone modifications have been identified that have important roles for erythroid development, future studies targeting critical loci could help provide ideal strategies for treating anemia. There are also numerous research efforts focused on the development of inhibitors of various histone modifying enzymes in order to improve different biological effects. However, histone modifying enzymes as well as the various complexes formed have a wide range of substrates and roles, and future studies are still needed to further distinguish their roles as catalytic enzymes and scaffold proteins.

**Table 1.** Histone methylation and their enzymes involved in erythropoiesis.

Methylation site	Methyltransferases			Demethylases		
	Protein	Roles in erythropoiesis	References	Protein	Roles in erythropoiesis	References
H3K4	KMT2A (MLL1)	Regulates hematopoietic proliferation and differentiation	[16,17,21]	LSD1	Silences the expression of γ-globin during adult erythropoiesis; involves in hemoglobin synthesis and erythroid differentiation associated with GATA1, TAL1	[30,31,32, 33]
	KMT2B (MLL2)	Regulates the expression of β-Globin	[23, 24]			
	SETD1A	Maintain HSCs proliferation and differentiation; regulates erythroid differentiation	[26]			
	SETD1B	Regulates the expression of erythroid lineage specific transcription factors(GATA1, KLF1)	[27]			
H3K9	SUV39H1	Regulates senescence of hematopoietic stem cells; overexpression of SUV39H1 induced erythroblasts immortalize and abnormal erythroblasts differentiation	[37, 38,39]	PHF2	Influences megakaryocytic and erythroid differentiation	[45]
	SETDB1	Regulates hematopoietic lineage differentiation; maintains the differentiation of HSCs into macrophages and erythroid lineages	[40, 41]			
	G9A/GLP	Regulate the silencing of *HBE1*, *HBG1* and *HBG2* genes	[42–44]			
H3K27	EZH2	Regulates the differentiation and growth of erythroid progenitor cells; maintains the enucleation in terminal erythroid differentiation	[53]	UTX	Protects the function of HSCs	[56]
				JMJD3	Be associated with malignant hematopoiesis	[57]
H3K36	SETD2	Maintains the function of HSCs; Regulates the expression of erythroid lineage regulators (GATA1, KLF1, GFI1B, and ZFPM1)	[59,61,62]	KDM2B	Be essential for maintenance and lineage commitment of HSPCs	[66]
	NSD1	Regulates maturation of terminal erythrocytes	[63]			
H3K79	DOT1L	Regulates the switch of GATA1 and GATA2, Maintains primitive erythropoiesis	[72]			
H4K20	SETD8	Promotes proerythroblasts into terminal erythroid differentiation stage; promotes enucleation and hemogobin accumulation	[81, 82]	SUV420H1 SUV420H2	Promotes chromatin condensation; inhibits the ε-globin gene in primary erythroid progenitor cells	[84,86]
H3R2	PRMT6	Inhibits the expression of various erythroid-specific genes (*AHSP*, *ALAS2*, *GYPA* and *KLF1*);	[88]			
H4R3	PRMT5	Promotes erythroid differentiation	[99]			
	PRMT1	Regulates the self-renewal ability of HSPCs; promotes terminal erythroid differentiation; regulates the expression of β-globin	[100,101]			

**Table 2.** Histone acetylation and their enzymes involved in erythropoiesis.

Acetylation site	Acetyltransferases			Deacetylases		
	Protein	Roles in erythropoiesis	References	Protein	Roles in erythropoiesis	References
H3K9	KAT2A	Regulates erythroid progenitor development and terminal erythroid differentiation; regulates the expression of EPOR	[112, 113]	SIRT6	Plays an important role in the maturation and aging of erythrocytes	[116]
H3K14	HBO1 BRD1	Maintain the fouction of HSCs; regulates erythroid progenitor differentiation; prevents apoptosis of erythroblasts in fetal liver	[117]			
	KAT6A	Regulates proliferation of early hematopoietic precursors	[119]			
H3K27	P300	Regulates HSC differentiation	[123]	HDAC3	Regulates HSC quiescence	[128, 129]
	CBP	Regulates HSC self-renewal and inhibits myeloid differentiation	[124]			
H3K56	GCN5	Regulates the expression globin and erythroid-specific genes	[132]	SIRT1	Plays an important role in myeloid and lymphoid specification; activates the expression of γ-globin	[133,134]
				SIRT6	Plays an important role in HSC homeostasis	[135,136]
H4K12				HDAC5	Regulates chromatin condensation and enucleation in terminal erythriod differentiation	[6]
H4K16	MOF	Maintenance of HSC self-renewal capacity; impacts early erythroid development and terminal differentiation	[154, 155,156]			

Human hemoglobin is dynamic throughout development, with fetal hemoglobin (HbF) being the main component during fetal development, consisting of two α-globin and two γ-globin chains. As oxygen supply increases after birth, fetal hemoglobin gradually gives way to adult hemoglobin (HbA), which consists of two α-globin and two β-globin chains. This transition is achieved through gene expression regulation, involving precise regulation by various transcription factors and signaling pathways. Abnormalities in adult β-globin lead to two types of anemia: β-thalassemia, which is mainly caused by the absence of β-globin, and sickle cell disease, which is mainly caused by mutations in the β-globin gene. Inducing the expression of γ-globin in adults can effectively ameliorate beta-hemoglobinopathies, β-thalassemia, and sickle cell disease. Numerous studies have indicated that histone modifications regulate the expression of globins. The methylation enzyme MLL2 of H3K4 regulates the expression of β-globin [30], while the demethylase LSD1 of H3K4 methylation inhibits the expression of γ-globin, and the inhibition of LSD1 can induce the expression of γ-globin [36,37]; PRMT1 regulates H3R4me at the β-globin locus, preventing the enrichment of H3K9me and H3K27me and promoting the expression of β-globin [105]; H3K9 demethylase G9A and GLP can regulate the silencing of *HBE1*, *HBG1* and *HBG2* genes [49]; many members of the histone deacetylase(HDAC) family can also regulate the expression of γ-globin, such as HDAC1, HDAC3 and HDAC9, which bind to the promoter region of the γ-globin gene and upregulates its gene expression [156,157]. The re-expression of γ-globin in adult erythrocytes mediated by histone modifications might be an effective strategy for alleviating β-thalassemia and sickle cell disease.

Currently, multiple histone deacetylase inhibitors (HDACi) hold promising therapeutic prospects for the treatment of thalassemia and sickle cell anemia. Vorinostat has been utilized in clinical trials targeting sickle cell disease (SCD) patients to reactivate HbF expression [174]. CT-101 demonstrated additional activity on HbF when combined with hydroxyurea (HU) in preclinical studies involving erythroid progenitor cells derived from SCD [175]. Sodium butyrate also possesses the ability to induce HbF, exhibiting a reduction in hospitalization rates, thereby establishing its advantage in SCD treatment [176]. MS-275 can also induce HbF production, and its analog MD-48 demonstrates the highest activity [177]. In addition, IOX1, a histone demethylase inhibitor, downregulates the expression of α- and α-like globins without affecting β-like globin expression or erythroid differentiation, potentially becoming a crucial component in the treatment of β-thalassemia [178]. Preclinical studies using SIRT1 activator molecules, such as SRT2104 or SRT1720, have shown reactivation of the γ-globin gene [139]. RN-1, a potent and irreversible LSD1 inhibitor [179], or FTX-6058, an inhibitor of EED [180], as well as UNC0638, a selective inhibitor of EHMT1/2, have been used in preclinical animal studies to trigger HbF expression and may be further developed [181].

Blood transfusion is an essential intervention for managing significant blood loss; however, the persistent shortage of supply in blood banks has consistently impacted the efficacy of transfusion therapies. The pursuit of *ex vivo* hematopoiesis is critically important. Nonetheless, a major challenge currently hindering this process is the blockade of red cell enucleation, which obstructs the generation of mature and functional erythrocytes. Research indicates that histone modification regulatory proteins play a crucial role in modulating red cell enucleation, potentially serving as effective mediators to enhance both red cell maturation and *ex vivo* hematopoiesis. The methyltransferase EZH2 independently sustains the enucleation of terminal erythrocytes during terminal erythropoiesis [58], whereas deficiencies in SETD2 and SETD8 result in diminished de-nucleation efficiency among Ortho-Es [65,87]. Additionally, the deacetylase HDAC5 significantly influences chromatin condensation and enucleation processes [12].

In this review, we systematically explore the regulatory roles and molecular mechanisms of histone methylation and acetylation modifications in hematopoietic and erythroid cell development. These epigenetic insights not only deepen our understanding of the fundamental biology underlying erythropoiesis but also provide critical perspectives on the pathogenesis of erythrocyte-related disorders. Moreover, these findings offer valuable implications for developing innovative strategies to optimize erythropoiesis in both *in vitro* and *in vivo* settings, with significant potential applications in transfusion medicine.

## Supplementary Material

Figure legend.docx

## Data Availability

All figures and tables are original and are not taken from other publications. Data sharing is not applicable to this article, as no new data were created or analysed in this study.

## References

[1] Li J, Hale J, Bhagia P, et al. Isolation and transcriptome analyses of human erythroid progenitors: BFU-E and CFU-E. Blood. 2014;124(24):3636–3645. doi: 10.1182/blood-2014-07-588806. 25339359 PMC4256913

[2] Ney PA. Normal and disordered reticulocyte maturation. Curr Opin Hematol. 2011;18(3):152–157. doi: 10.1097/MOH.0b013e328345213e. 21423015 PMC3157046

[3] Caulier A, Sankaran VG. Molecular and cellular mechanisms that regulate human erythropoiesis. Blood. 2022;140(12):1451–1451. doi: 10.1182/blood.2022017227. PMC9029096 34936695

[4] An XL, Schulz VP, Li J, et al. Global transcriptome analyses of human and murine terminal erythroid differentiation. Blood. 2014;123(22):3466–3477. doi: 10.1182/blood-2014-01-548305. 24637361 PMC4041167

[5] Allshire RC, Madhani HD. Ten principles of heterochromatin formation and function. Nat Rev Mol Cell Biol. 2018;19(4):229–244. doi: 10.1038/nrm.2017.119. 29235574 PMC6822695

[6] Falk M, Feodorova Y, Naumova N, et al. Heterochromatin drives compartmentalization of inverted and conventional nuclei. Nature. 2019;570(7761):395–399. doi: 10.1038/s41586-019-1275-3. 31168090 PMC7206897

[7] Buchwalter A, Kaneshiro JM, Hetzer MW. Coaching from the sidelines: the nuclear periphery in genome regulation. Nat Rev Genet. 2019;20(1):39–50. doi: 10.1038/s41576-018-0063-5. 30356165 PMC6355253

[8] Padeken J, Methot SP, Gasser SM. Establishment of H3K9-methylated heterochromatin and its functions in tissue differentiation and maintenance. Nat Rev Mol Cell Biol. 2022;23(9):623–640. doi: 10.1038/s41580-022-00483-w. 35562425 PMC9099300

[9] Mei Y, Liu YJ, Ji P. Understanding terminal erythropoiesis: an update on chromatin condensation, enucleation, and reticulocyte maturation. Blood Rev. 2021;46:100740. doi: 10.1016/j.blre.2020.100740. 32798012

[10] Qu XL, Zhang SJ, Wang SH, et al. TET2 deficiency leads to stem cell factor-dependent clonal expansion of ­dysfunctional erythroid progenitors. Blood. 2018;132(22):2406–2417. doi: 10.1182/blood-2018-05-853291. 30254129 PMC6265651

[11] Yang QQ, Chen LX, Zhang HC, et al. DNMT1 regulates human erythropoiesis by modulating cell cycle and endoplasmic reticulum stress in a stage-specific manner. Cell Death Differ. 2024;31(8):999–1012. doi: 10.1038/s41418-024-01305-6. 38719927 PMC11303534

[12] Wang YM, Li W, Schulz VP, et al. Impairment of human terminal erythroid differentiation by histone deacetylase 5 deficiency. Blood. 2021;138(17):1615–1627. doi: 10.1182/blood.2020007401. 34036344 PMC8554652

[13] Paralkar VR, Mishra T, Luan J, et al. Lineage and species-specific long noncoding RNAs during erythro-megakaryocytic development. Blood. 2014;123(12):1927–1937. doi: 10.1182/blood-2013-12-544494. 24497530 PMC3962165

[14] Li YJ, Ge KX, Li TT, et al. The engagement of histone lysine methyltransferases with nucleosomes: structural basis, regulatory mechanisms, and therapeutic implications. Protein Cell. 2023;14(3):165–179. doi: 10.1093/procel/pwac032. 37051671 PMC10098044

[15] Jambhekar A, Dhall A, Shi Y. Roles and regulation of histone methylation in animal development. Nat Rev Mol Cell Biol. 2019;20(10):625–641. doi: 10.1038/s41580-019-0151-1. 31267065 PMC6774358

[16] Bannister AJ, Kouzarides T. Reversing histone methylation. Nature. 2005;436(7054):1103–1106. doi: 10.1038/nature04048. 16121170

[17] Yang C, Zhang JY, Ma YK, et al. Histone methyltransferase and drug resistance in cancers. J Exp Clin Canc Res. 2020;39(1):173. doi: 10.1186/s13046-020-01682-z. PMC7455899 32859239

[18] Kooistra SM, Helin K. Molecular mechanisms and potential functions of histone demethylases. Nat Rev Mol Cell Biol. 2012;13(5):297–311. doi: 10.1038/nrm3327. 22473470

[19] Andricovich J, Kai Y, Tzatsos A. Lysine-specific histone demethylases in normal and malignant hematopoiesis. Exp Hematol. 2016;44(9):778–782. doi: 10.1016/j.exphem.2016.05.006. 27208808 PMC5100667

[20] Yang WW, Ernst P. Distinct functions of histone H3, lysine 4 methyltransferases in normal and malignant hematopoiesis. Curr Opin Hematol. 2017;24(4):322–328. doi: 10.1097/Moh.0000000000000346. 28375985 PMC5603181

[21] Yu HM, Lesch BJ. Functional roles of H3K4 methylation in transcriptional regulation. Mol Cell Biol. 2024;44(11):505–515. doi: 10.1080/10985549.2024.2388254. 39155435 PMC11529435

[22] Li YJ, Han JM, Zhang YB, et al. Structural basis for activity regulation of MLL family methyltransferases. Nature. 2016;530(7591):447–452. doi: 10.1038/nature16952. 26886794 PMC5125619

[23] Cai SF, Zhu Q, Guo CL, et al. MLL1 promotes myogenesis by epigenetically regulating. Cell Proliferat. 2020;53(2):e12744. doi: 10.1111/cpr.12744. PMC7046306 31840352

[24] Ernst P, Fisher JK, Avery W, et al. Definitive hematopoiesis requires the mixed-lineage leukemia gene. Dev Cell. 2004;6(3):437–443. doi: 10.1016/S1534-5807(04)00061-9. 15030765

[25] Hess JL, Yu BD, Li B, et al. Defects in yolk sac hematopoiesis in Mll-null embryos. Blood. 1997;90(5):1799–1806. doi: 10.1182/blood.V90.5.1799. 9292512

[26] McMahon KA, Hiew SYL, Hadjur S, et al. MII has a critical role in fetal and adult hematopoietic stem cell self-renewal. Cell Stem Cell. 2007;1(3):338–345. doi: 10.1016/j.stem.2007.07.002. 18371367

[27] Mishra BP, Zaffuto KM, Artinger EL, et al. The histone methyltransferase activity of MLL1 is dispensable for hematopoiesis and leukemogenesis. Cell Rep. 2014;7(4):1239–1247. doi: 10.1016/j.celrep.2014.04.015. 24813891 PMC4120120

[28] Klonou A, Chlamydas S, Piperi C. Structure, activity and function of the MLL2 (KMT2B) protein lysine methyltransferase. Life-Basel. 2021;11(8):823. doi: 10.3390/life11080823. 34440566 PMC8401916

[29] Austenaa L, Barozzi I, Chronowska A, et al. The histone methyltransferase Wbp7 controls macrophage function through GPI glycolipid anchor synthesis. Immunity. 2012;36(4):572–585. doi: 10.1016/j.immuni.2012.02.016. 22483804

[30] Demers C, Chaturvedi CP, Ranish JA, et al. Activator-mediated recruitment of the MLL2 methyltransferase complex to the β-globin locus. Mol Cell. 2007;27(4):573–584. doi: 10.1016/j.molcel.2007.06.022. 17707229 PMC2034342

[31] Arndt K, Kranz A, Fohgrub J, et al. SETD1A protects HSCs from activation-induced functional decline in vivo. Blood. 2018;131(12):1311–1324. doi: 10.1182/blood-2017-09-806844. 29348130

[32] Li Y, Schulz VP, Deng CW, et al. Setd1a and NURF mediate chromatin dynamics and gene regulation during erythroid lineage commitment and differentiation. Nucleic Acids Res. 2016;44(15):7173–7188. doi: 10.1093/nar/gkw327. 27141965 PMC5009724

[33] Schmidt K, Zhang QY, Tasdogan A, et al. The H3K4 methyltransferase Setd1b is essential for hematopoietic stem and progenitor cell homeostasis in mice. Elife. 2018;7:e27157. doi: 10.7554/eLife.27157. 29916805 PMC6025962

[34] Kerenyi MA, Shao Z, Hsu YJ, et al. Histone demethylase Lsd1 represses hematopoietic stem and progenitor cell signatures during blood cell maturation. Elife. 2013;2:e00633. doi: 10.7554/eLife.00633. 23795291 PMC3687337

[35] Zhao AL, Zhou H, Yang JR, et al. Epigenetic regulation in hematopoiesis and its implications in the targeted therapy of hematologic malignancies. Sig Transduct Target Ther. 2023;8(1):71. doi: 10.1038/s41392-023-01342-6. PMC9935927 36797244

[36] Yu L, Myers G, Ku CJ, et al. An erythroid-to-myeloid cell fate conversion is elicited by LSD1 inactivation. Blood. 2021;138(18):1691–1704. doi: 10.1182/blood.2021011682. 34324630 PMC8569417

[37] Shi LH, Cui SY, Engel JD, et al. Lysine-specific demethylase 1 is a therapeutic target for fetal hemoglobin induction. Nat Med. 2013;19(3):291–294. doi: 10.1038/nm.3101. 23416702 PMC5512162

[38] Kohrogi K, Hino S, Sakamoto A, et al. LSD1 defines erythroleukemia metabolism by controlling the lineage-specific transcription factors GATA1 and C/EBPα. Blood Adv. 2021;5(9):2305–2318. doi: 10.1182/bloodadvances.2020003521. 33929501 PMC8114557

[39] Hu X, Li XG, Valverde K, et al. LSD1-mediated epigenetic modification is required for TAL1 function and hematopoiesis. Proc Natl Acad Sci USA. 2009;106(25):10141–10146. doi: 10.1073/pnas.0900437106. 19497860 PMC2700898

[40] Wang JX, Saijo K, Skola D, et al. Histone demethylase LSD1 regulates hematopoietic stem cells homeostasis and protects from death by endotoxic shock. Proc Natl Acad Sci USA. 2018;115(2):E244–E252. doi: 10.1073/pnas.1718759114. 29263096 PMC5777077

[41] Zhang SJ, Liu MH, Yao YF, et al. Targeting LSD1 for acute myeloid leukemia (AML) treatment. Pharmacol Res. 2021;164:105335. doi: 10.1016/j.phrs.2020.105335. 33285227

[42] Maeda R, Tachibana M. HP1 maintains protein stability of H3K9 methyltransferases and demethylases. EMBO Rep. 2022;23(4):e53581. doi: 10.15252/embr.202153581. 35166421 PMC8982598

[43] Keenan CR, Iannarella N, Naselli G, et al. Extreme disruption of heterochromatin is required for accelerated hematopoietic aging. Blood. 2020;135(23):2049–2058. doi: 10.1182/blood.2019002990. 32305044

[44] Czvitkovich S, Sauer S, Peters AHFM, et al. Over-expression of the SUV39H1 histone methyltransferase induces altered proliferation and differentiation in transgenic mice. Mech Dev. 2001;107(1–2):141–153. doi: 10.1016/S0925-4773(01)00464-6. 11520670

[45] Wu JX, Li J, Chen K, et al. Atf7ip and Setdb1 interaction orchestrates the hematopoietic stem and progenitor cell state with diverse lineage differentiation. Proc Natl Acad Sci USA. 2023;120(1):e2209062120. doi: 10.1073/pnas.2209062120. 36577070 PMC9910619

[46] Koide S, Oshima M, Takubo K, et al. Setdb1 maintains hematopoietic stem and progenitor cells by restricting the ectopic activation of nonhematopoietic genes. Blood. 2016;128(5):638–649. doi: 10.1182/blood-2016-01-694810. 27301860

[47] Renneville A, Van Galen P, Canver MC, et al. EHMT1 and EHMT2 inhibition induces fetal hemoglobin expression. Blood. 2015;126(16):1930–1939. doi: 10.1182/blood-2015-06-649087. 26320100 PMC4608240

[48] Chen XJ, Skutt-Kakaria K, Davison J, et al. G9a/GLP-dependent histone H3K9me2 patterning during human hematopoietic stem cell lineage commitment. Genes Dev. 2012;26(22):2499–2511. doi: 10.1101/gad.200329.112. 23105005 PMC3505820

[49] Krivega I, Byrnes C, de Vasconcellos JF, et al. Inhibition of G9a methyltransferase stimulates fetal hemoglobin production by facilitating LCR/γ-globin looping. Blood. 2015;126(5):665–672. doi: 10.1182/blood-2015-02-629972. 25979948 PMC4520881

[50] Yang JC, Ma J, Xiong Y, et al. Epigenetic regulation of megakaryocytic and erythroid differentiation by PHF2 histone demethylase. J Cell Physiol. 2018;233(9):6841–6852. doi: 10.1002/jcp.26438. 29336484 PMC13482482

[51] Ferrari KJ, Scelfo A, Jammula S, et al. Polycomb-dependent H3K27me1 and H3K27me2 regulate active transcription and enhancer fidelity. Mol Cell. 2014;53(1):49–62. doi: 10.1016/j.molcel.2013.10.030. 24289921

[52] Hidalgo I, Herrera-Merchan A, Ligos JM, et al. Ezh1 is required for hematopoietic stem cell maintenance and prevents senescence-like cell cycle arrest. Cell Stem Cell. 2012;11(5):649–662. doi: 10.1016/j.stem.2012.08.001. 23122289

[53] Komrokji R, Aguirre LE, Al Ali N, et al. U2AF1 and EZH2 mutations are associated with nonimmune hemolytic anemia in myelodysplastic syndromes. Blood Adv. 2023;7(1):1–8. doi: 10.1182/bloodadvances.2022007504. 36129843 PMC9813529

[54] Mochizuki-Kashio M. Dependency on the polycomb gene distinguishes fetal from adult hematopoietic stem cells (vol 118, pg 6553, 2011). Blood. 2012;120(4):924–924. 10.1182/blood-2011-03-340554 22042701

[55] Gu ZM, Liu YX, Cai F, et al. Loss of EZH2 reprograms BCAA metabolism to drive leukemic transformation. Cancer Discov. 2019;9(9):1228–1247. doi: 10.1158/2159-8290.Cd-19-0152. 31189531 PMC6726547

[56] Mazzi S, Dessen P, Vieira M, et al. Dual role of EZH2 in megakaryocyte differentiation. Blood. 2021;138(17):1603–1614. doi: 10.1182/blood.2019004638. 34115825 PMC8554649

[57] Neo WH, Booth CAG, Azzoni E, et al. Cell-extrinsic hematopoietic impact of Ezh2 inactivation in fetal liver endothelial cells. Blood. 2018;131(20):2223–2234. doi: 10.1182/blood-2017-10-811455. 29555646 PMC5960588

[58] Li MJ, Liu DH, Xue FM, et al. Stage-specific dual function: EZH2 regulates human erythropoiesis by eliciting histone and non-histone methylation. Haematologica. 2023;108(9):2487–2502. doi: 10.3324/haematol.2022.282016. 37021526 PMC10483364

[59] Zhong YB, Ye Q, Chen CY, et al. Ezh2 promotes clock function and hematopoiesis independent of histone methyltransferase activity in zebrafish. Nucleic Acids Res. 2018;46(7):3382–3399. doi: 10.1093/nar/gky101. 29447387 PMC5909462

[60] Hu P, Nebreda AR, Hanenberg H, et al. P38α/JNK signaling restrains erythropoiesis by suppressing Ezh2-mediated epigenetic silencing of Bim. Nat Commun. 2018;9(1):3518. doi: 10.1038/s41467-018-05955-2. 30158520 PMC6115418

[61] Sera Y, Nakata Y, Ueda T, et al. UTX maintains the functional integrity of the murine hematopoietic system by globally regulating aging-associated genes. Blood. 2021;137(7):908–922. doi: 10.1182/blood.2019001044. 33174606 PMC7918186

[62] Yu SH, Zhu KY, Chen J, et al. JMJD3 facilitates C/EBPβ-centered transcriptional program to exert oncorepressor activity in AML. Nat Commun. 2018;9(1):3369. doi: 10.1038/s41467-018-05548-z. 30135572 PMC6105679

[63] Shpargel KB, Sengoku T, Yokoyama S, et al. UTX and UTY demonstrate histone demethylase-independent function in mouse embryonic development. PLoS Genet. 2012;8(9):e1002964. doi: 10.1371/journal.pgen.1002964. 23028370 PMC3459986

[64] Brumbaugh J, Kim IS, Ji F, et al. Inducible histone K-to-M mutations are dynamic tools to probe the physiological role of site-specific histone methylation in vitro and in vivo. Nat Cell Biol. 2019;21(11):1449–1461. doi: 10.1038/s41556-019-0403-5. 31659274 PMC6858577

[65] Li YL, Tang HY, Chen FL, et al. SETD2 is essential for terminal differentiation of erythroblasts during fetal erythropoiesis. Biochem Biophys Res Commun. 2021;552:98–105. doi: 10.1016/j.bbrc.2021.03.040. 33743353

[66] Zhang YL, Sun JW, Xie YY, et al. Setd2 deficiency impairs hematopoietic stem cell self-renewal and causes malignant transformation. Cell Res. 2018;28(4):476–490. doi: 10.1038/s41422-018-0015-9. 29531312 PMC5939047

[67] Zhou YL, Yan XM, Feng XM, et al. Setd2 regulates quiescence and differentiation of adult hematopoietic stem cells by restricting RNA polymerase II elongation. Haematologica. 2018;103(7):1110–1123. doi: 10.3324/haematol.2018.187708. 29650642 PMC6029524

[68] Leonards K, Almosailleakh M, Tauchmann S, et al. Nuclear interacting SET domain protein 1 inactivation impairs GATA1-regulated erythroid differentiation and causes erythroleukemia. Nat Commun. 2020;11(1):2807. doi: 10.1038/s41467-020-16179-8. 32533074 PMC7293310

[69] Tsukada Y, Fang J, Erdjument-Bromage H, et al. Histone demethylation by a family of JmjC domain-containing proteins. Nature. 2006;439(7078):811–816. doi: 10.1038/nature04433. 16362057

[70] Klose RJ, Kallin EM, Zhang Y. JmjC-domain-containing proteins and histone demethylation. Nat Rev Genet. 2006;7(9):715–727. doi: 10.1038/nrg1945. 16983801

[71] Andricovich J, Kai Y, Peng WQ, et al. Histone demethylase KDM2B regulates lineage commitment in normal and malignant hematopoiesis. J Clin Invest. 2016;126(3):905–920. doi: 10.1172/Jci84014. 26808549 PMC4767361

[72] Deshpande AJ, Deshpande A, Sinha AU, et al. AF10 regulates progressive H3K79 methylation and HOX gene expression in diverse AML subtypes. Cancer Cell. 2014;26(6):896–908. doi: 10.1016/j.ccell.2014.10.009. 25464900 PMC4291116

[73] Haladyna JN, Yamauchi T, Neff T, et al. Epigenetic modifiers in normal and malignant hematopoiesis. Epigenomics-Uk. 2015;7(2):301–320. doi: 10.2217/Epi.14.88. 25942537

[74] Assi R, Cherifi C, Cornelis FMF, et al. Inhibition of KDM7A/B histone demethylases restores H3K79 methylation and protects against osteoarthritis. Ann Rheum Dis. 2023;82(7):963–973. doi: 10.1136/ard-2022-223789. 36927643

[75] Kang JY, Park JW, Hahm JY, et al. Histone H3K79 demethylation by KDM2B facilitates proper DNA replication through PCNA dissociation from chromatin. Cell Proliferat. 2020;53(11):e12920. doi: 10.1111/cpr.12920. PMC7653264 33029857

[76] Feng Y, Yang YP, Ortega MM, et al. Early mammalian erythropoiesis requires the Dot1L methyltransferase. Blood. 2010;116(22):4483–4491. doi: 10.1182/blood-2010-03-276501. 20798234 PMC3321834

[77] Malcom CA, Ratri A, Piasecka-Srader J, et al. Primitive erythropoiesis in the mouse is independent of DOT1L methyltransferase activity. Front Cell Dev Biol. 2022;9:813503. doi: 10.3389/fcell.2021.813503. 35111761 PMC8802720

[78] Borosha S, Ratri A, Ghosh S, et al. DOT1L mediated gene repression in extensively self-renewing erythroblasts. Front Genet. 2022;13:828086. doi: 10.3389/fgene.2022.828086. 35401699 PMC8984088

[79] Grigsby SM, Friedman A, Chase J, et al. Elucidating the importance of DOT1L recruitment in MLL-AF9 leukemia and hematopoiesis. Cancers (Basel). 2021;13(4):642. doi: 10.3390/cancers13040642. 33562706 PMC7914713

[80] Nguyen AT, He J, Taranova O, et al. Essential role of DOT1L in maintaining normal adult hematopoiesis. Cell Res. 2011;21(9):1370–1373. doi: 10.1038/cr.2011.115. 21769133 PMC3166961

[81] Beck DB, Oda H, Shen SS, et al. PR-Set7 and H4K20me1: at the crossroads of genome integrity, cell cycle, chromosome condensation, and transcription. Genes Dev. 2012;26(4):325–337. doi: 10.1101/gad.177444.111. 22345514 PMC3289880

[82] Oda H, Okamoto I, Murphy N, et al. Monomethylation of histone H4-Lysine 20 is involved in chromosome structure and stability and is essential for mouse development. Mol Cell Biol. 2009;29(8):2278–2295. doi: 10.1128/Mcb.01768-08. 19223465 PMC2663305

[83] Milite C, Feoli A, Viviano M, et al. The emerging role of lysine methyltransferase SETD8 in human diseases. Clin Epigenetics. 2016;8:102. doi: 10.1186/s13148-016-0268-4. 27688818 PMC5034662

[84] Malik J, Lillis JA, Couch T, et al. The methyltransferase Setd8 is essential for erythroblast survival and maturation. Cell Rep. 2017;21(9):2376–2383. doi: 10.1016/j.celrep.2017.11.011. 29186677 PMC7748363

[85] Nishioka K, Rice JC, Sarma K, et al. PR-Set7 is a nucleosome-specific methyltransferase that modifies lysine 20 of histone H4 and is associated with silent chromatin. Mol Cell. 2002;9(6):1201–1213. doi: 10.1016/S1097-2765(02)00548-8. 12086618

[86] DeVilbiss AW, Sanalkumar R, Hall BDR, et al. Epigenetic determinants of erythropoiesis: role of the histone methyltransferase SetD8 in promoting erythroid cell maturation and survival. Mol Cell Biol. 2015;35(12):2073–2087. doi: 10.1128/Mcb.01422-14. 25855754 PMC4438249

[87] Malik J, Getman M, Steiner LA. Histone methyltransferase Setd8 represses Gata2 expression and regulates erythroid maturation. Mol Cell Biol. 2015;35(12):2059–2072. doi: 10.1128/Mcb.01413-14. 25848090 PMC4438238

[88] Myers JA, Couch T, Murphy Z, et al. The histone methyltransferase Setd8 alters the chromatin landscape and regulates the expression of key transcription factors during erythroid differentiation. Epigenet Chromatin. 2020;13(1):16. doi: 10.1186/s13072-020-00337-9. PMC7075014 32178723

[89] Schotta G, Sengupta R, Kubicek S, et al. A chromatin-wide transition to H4K20 monomethylation impairs genome integrity and programmed DNA rearrangements in the mouse. Genes Dev. 2008;22(15):2048–2061. doi: 10.1101/gad.476008. 18676810 PMC2492754

[90] Papagiannopoulos CI, Theodoroula NF, Kyritsis KA, et al. The histone methyltransferase inhibitor A-366 enhances hemoglobin expression in erythroleukemia cells upon co-exposure with chemical inducers in culture. J Biol Res (Thessalon). 2021;28(1):2. doi: 10.1186/s40709-020-00132-3. 33407944 PMC7788816

[91] Wang YD, Rank G, Li ZC, et al. ε-globin expression is regulated by SUV4-20h1. Haematologica. 2016;101(5):e168–e172. doi: 10.3324/haematol.2015.139980. 26802048 PMC5004379

[92] Gillinder KR, Tuckey H, Bell CC, et al. Direct targets of pSTAT5 signalling in erythropoiesis. PLoS One. 2017;12(7):e0180922. doi: 10.1371/journal.pone.0180922. 28732065 PMC5521770

[93] Herkt SC, Kuvardina ON, Herglotz J, et al. Protein arginine methyltransferase 6 controls erythroid gene expression and differentiation of human CD34+ progenitor cells. Haematologica. 2018;103(1):18–29. doi: 10.3324/haematol.2017.174516. 29025910 PMC5777187

[94] Stein C, Nötzold RR, Riedl S, et al. The arginine methyltransferase PRMT6 cooperates with polycomb proteins in regulating HOXA gene expression. PLoS One. 2016;11(2):e0148892. doi: 10.1371/journal.pone.0148892. 26848759 PMC4746130

[95] Stein C, Riedl S, Rüthnick D, et al. The arginine methyltransferase PRMT6 regulates cell proliferation and senescence through transcriptional repression of tumor suppressor genes. Nucleic Acids Res. 2012;40(19):9522–9533. doi: 10.1093/nar/gks767. 22904088 PMC3479209

[96] Phalke S, Mzoughi S, Bezzi M, et al. p53-independent regulation of p21Waf1/Cip1 expression and senescence by PRMT6. Nucleic Acids Res. 2012;40(19):9534–9542. doi: 10.1093/nar/gks858. 22987071 PMC3479215

[97] Wang XL, Huang Y, Zhao J, et al. Suppression of PRMT6-mediated arginine methylation of p16 protein potentiates its ability to arrest A549 cell proliferation. Int J Biochem Cell Biol. 2012;44(12):2333–2341. doi: 10.1016/j.biocel.2012.09.015. 23032699

[98] Liu C, Zou WY, Nie DN, et al. Loss of PRMT7 reprograms glycine metabolism to selectively eradicate leukemia stem cells in CML. Cell Metab. 2022;34(6):818–835.e7. doi: 10.1016/j.cmet.2022.04.004. 35508169

[99] Greenblatt SM, Man N, Hamard PJ, et al. CARM1 is essential for myeloid leukemogenesis but dispensable for normal hematopoiesis. Cancer Cell. 2018;33(6):1111–1127.e5. doi: 10.1016/j.ccell.2018.05.007. 29894694 PMC6191185

[100] Karkhanis V, Hu YJ, Baiocchi RA, et al. Versatility of PRMT5-induced methylation in growth control and development. Trends Biochem Sci. 2011;36(12):633–641. doi: 10.1016/j.tibs.2011.09.001. 21975038 PMC3225484

[101] Fabbrizio E, El Messaoudi S, Polanowska J, et al. Negative regulation of transcription by the type II arginine methyltransferase PRMT5. EMBO Rep. 2002;3(7):641–645. doi: 10.1093/embo-reports/kvf136. 12101096 PMC1084190

[102] Wang L, Pal S, Sif S. Protein arginine methyltransferase 5 suppresses the transcription of the RB family of tumor suppressors in leukemia and lymphoma cells. Mol Cell Biol. 2008;28(20):6262–6277. doi: 10.1128/Mcb.00923-08. 18694959 PMC2577430

[103] Le Guezennec X, Vermeulen M, Brinkman AB, et al. MBD2/NuRD and MBD3/NuRD, two distinct complexes with different biochemical and functional properties. Mol Cell Biol. 2006;26(3):843–851. doi: 10.1128/Mcb.26.3.843-851.2006. 16428440 PMC1347035

[104] Liu F, Cheng GY, Hamard PJ, et al. Arginine methyltransferase PRMT5 is essential for sustaining normal adult hematopoiesis. J Clin Invest. 2015;125(9):3532–3544. doi: 10.1172/Jci81749. 26258414 PMC4588241

[105] Huang SM, Litt M, Felsenfeld G. Methylation of histone H4 by arginine methyltransferase PRMT1 is essential in vivo for many subsequent histone modifications. Genes Dev. 2005;19(16):1885–1893. doi: 10.1101/gad.1333905. 16103216 PMC1186188

[106] Zhu L, He X, Dong HJ, et al. Protein arginine methyltransferase 1 is required for maintenance of normal adult hematopoiesis. Int J Biol Sci. 2019;15(13):2763–2773. doi: 10.7150/ijbs.38859. 31853216 PMC6909962

[107] Sun XJ, Man N, Tan YR, et al. The role of histone acetyltransferases in normal and malignant hematopoiesis. Front Oncol. 2015;5:108. doi: 10.3389/fonc.2015.00108. 26075180 PMC4443728

[108] Wang P, Wang Z, Liu J. Role of HDACs in normal and malignant hematopoiesis. Mol Cancer. 2020;19(1):5. doi: 10.1186/s12943-019-1127-7. 31910827 PMC6945581

[109] Li YX, Seto E. HDACs and HDAC inhibitors in cancer development and therapy. Csh Perspect Med. 2016;6(10):a026831. doi: 10.1101/cshperspect.a026831. PMC5046688 27599530

[110] Roth SY, Denu JM, Allis CD. Histone acetyltransferases. Annu Rev Biochem. 2001;70(1):81–120. doi: 10.1146/annurev.biochem.70.1.81. 11395403

[111] Grozinger CM, Hassig CA, Schreiber SL. Three proteins define a class of human histone deacetylases related to yeast Hda1p. Proc Natl Acad Sci USA. 1999;96(9):4868–4873. doi: 10.1073/pnas.96.9.4868. 10220385 PMC21783

[112] Blander G, Guarente L. The Sir2 family of protein deacetylases. Annu Rev Biochem. 2004;73(1):417–435. doi: 10.1146/annurev.biochem.73.011303.073651. 15189148

[113] Vong P, Ouled-Haddou H, Garçon L. Histone deacetylases function in the control of early hematopoiesis and erythropoiesis. Int J Mol Sci. 2022;23(17):9790. doi: 10.3390/ijms23179790. 36077192 PMC9456231

[114] Karmodiya K, Krebs AR, Oulad-Abdelghani M, et al. H3K9 and H3K14 acetylation co-occur at many gene regulatory elements, while H3K14ac marks a subset of inactive inducible promoters in mouse embryonic stem cells. BMC Genomics. 2012;13(1):424. doi: 10.1186/1471-2164-13-424. 22920947 PMC3473242

[115] Guan HP, Wang P, Zhang P, et al. Diverse modes of H3K36me3-guided nucleosomal deacetylation by Rpd3S. Nature. 2023;620(7974):669–675. doi: 10.1038/s41586-023-06349-1. 37468628 PMC10432269

[116] Bonnet J, Wang CY, Baptista T, et al. The SAGA coactivator complex acts on the whole transcribed genome and is required for RNA polymerase II transcription. Genes Dev. 2014;28(18):1999–2012. doi: 10.1101/gad.250225.114. 25228644 PMC4173158

[117] Tusi BK, Wolock SL, Weinreb C, et al. Population snapshots predict early haematopoietic and erythroid hierarchies. Nature. 2018;555(7694):54–60. doi: 10.1038/nature25741. 29466336 PMC5899604

[118] Arede L, Foerner E, Wind S, et al. KAT2A complexes ATAC and SAGA play unique roles in cell maintenance and identity in hematopoiesis and leukemia. Blood Adv. 2022;6(1):165–180. doi: 10.1182/bloodadvances.2020002842. 34654054 PMC8753207

[119] Kouzarides T. Chromatin modifications and their function. Cell. 2007;128(4):693–705. doi: 10.1016/j.cell.2007.02.005. 17320507

[120] Kawahara TLA, Michishita E, Adler AS, et al. SIRT6 links histone H3 lysine 9 deacetylation to NF-κB-dependent gene expression and organismal life span. Cell. 2009;136(1):62–74. doi: 10.1016/j.cell.2008.10.052. 19135889 PMC2757125

[121] Chambers SM, Shaw CA, Gatza C, et al. Aging hematopoietic stem cells decline in function and exhibit epigenetic dysregulation. PLoS Biol. 2007;5(8):e201. doi: 10.1371/journal.pbio.0050201. 17676974 PMC1925137

[122] Mishima Y, Miyagi S, Saraya A, et al. The Hbo1-Brd1/Brpf2 complex is responsible for global acetylation of H3K14 and required for fetal liver erythropoiesis. Blood. 2011;118(9):2443–2453. doi: 10.1182/blood-2011-01-331892. 21753189

[123] Perez-Campo FM, Costa G, Lie-A-Ling M, et al. The MYSTerious MOZ, a histone acetyltransferase with a key role in haematopoiesis. Immunology. 2013;139(2):161–165. doi: 10.1111/imm.12072. 23347099 PMC3647182

[124] Perez-Campo FM, Borrow J, Kouskoff V, et al. The histone acetyl transferase activity of monocytic leukemia zinc finger is critical for the proliferation of hematopoietic precursors. Blood. 2009;113(20):4866–4874. doi: 10.1182/blood-2008-04-152017. 19264921 PMC2686138

[125] Sheikh BN, Yang YQ, Schreuder J, et al. MOZ (KAT6A) is essential for the maintenance of classically defined adult hematopoietic stem cells. Blood. 2016;128(19):2307–2318. doi: 10.1182/blood-2015-10-676072. 27663673

[126] Yang YQ, Kueh AJ, Grant ZL, et al. The histone lysine acetyltransferase HBO1 (KAT7) regulates hematopoietic stem cell quiescence and self-renewal. Blood. 2022;139(6):845–858. doi: 10.1182/blood.2021013954. 34724565

[127] Mah SYY, Vanyai HK, Yang YQ, et al. The chromatin reader protein ING5 is required for normal hematopoietic cell numbers in the fetal liver. Front Immunol. 2023;14:1119750. doi: 10.3389/fimmu.2023.1119750. 37275850 PMC10232820

[128] Rebel VI, Kung AL, Tanner EA, et al. Distinct roles for CREB-binding protein and p300 in hematopoietic stem cell self-renewal. Proc Natl Acad Sci USA. 2002;99(23):14789–14794. doi: 10.1073/pnas.232568499. 12397173 PMC137497

[129] Chan WI, Hannah RL, Dawson MA, et al. The transcriptional coactivator Cbp regulates self-renewal and differentiation in adult hematopoietic stem cells. Mol Cell Biol. 2011;31(24):5046–5060. doi: 10.1128/Mcb.05830-11. 22006020 PMC3233028

[130] Kimbrel EA, Lemieux ME, Xia XB, et al. Systematic in vivo structure-function analysis of p300 in hematopoiesis. Blood. 2009;114(23):4804–4812. doi: 10.1182/blood-2009-04-217794. 19822904

[131] Chi ZX, Chen S, Xu T, et al. Histone deacetylase 3 couples mitochondria to drive IL-1β-dependent inflammation by configuring fatty acid oxidation. Mol Cell. 2020;80(1):43–58.e7. doi: 10.1016/j.molcel.2020.08.015. 32937100

[132] Summers AR, Fischer MA, Stengel KR, et al. HDAC3 is essential for DNA replication in hematopoietic progenitor cells. J Clin Invest. 2013;123(7):3112–3123. doi: 10.1172/Jci60806. 23921131 PMC3696547

[133] Chen Z, Huo DW, Li L, et al. Nuclear DEK preserves hematopoietic stem cells potential via NCoR1/HDAC3-Akt1/2-mTOR axis. J Exp Med. 2021;218(5):e20201974. doi: 10.1084/jem.20201974. 33755722 PMC7992411

[134] Cabal-Hierro L, van Galen P, Prado MA, et al. Chromatin accessibility promotes hematopoietic and leukemia stem cell activity. Nat Commun. 2020;11(1):1406. doi: 10.1038/s41467-020-15221-z. 32179749 PMC7076002

[135] Topal S, Vasseur P, Radman-Livaja M, et al. Distinct transcriptional roles for histone H3-K56 acetylation during the cell cycle in Yeast. Nat Commun. 2019;10(1):4372. doi: 10.1038/s41467-019-12400-5. 31558720 PMC6763489

[136] Yuan J, Pu M, Zhang Z, et al. Histone H3-K56 acetylation is important for genomic stability in mammals. Cell Cycle. 2009;8(11):1747–1753. doi: 10.4161/cc.8.11.8620. 19411844 PMC2776713

[137] Woo AJ, Kim J, Xu J, et al. Role of ZBP-89 in human globin gene regulation and erythroid differentiation. Blood. 2011;118(13):3684–3693. doi: 10.1182/blood-2011-03-341446. 21828133 PMC3186340

[138] Rimmelé P, Bigarella CL, Liang R, et al. Aging-like phenotype and defective lineage specification in SIRT1-deleted hematopoietic stem and progenitor cells. Stem Cell Reports. 2014;3(1):44–59. doi: 10.1016/j.stemcr.2014.04.015. 25068121 PMC4110778

[139] Dai Y, Chen TW, Ijaz H, et al. SIRT_1_ activates the expression of fetal hemoglobin genes. Am J Hematol. 2017;92(11):1177–1186. doi: 10.1002/ajh.24879. 28776729 PMC5648331

[140] Luis TC, Naber BAE, Roozen PPC, et al. Canonical Wnt signaling regulates hematopoiesis in a dosage-dependent fashion. Cell Stem Cell. 2011;9(4):345–356. doi: 10.1016/j.stem.2011.07.017. 21982234

[141] Wang H, Diao DJ, Shi ZC, et al. SIRT6 controls hematopoietic stem cell homeostasis through epigenetic regulation of Wnt signaling. Cell Stem Cell. 2016;18(4):495–507. doi: 10.1016/j.stem.2016.03.005. 27058938

[142] Hua WK, Qi J, Cai Q, et al. HDAC8 regulates long-term hematopoietic stem-cell maintenance under stress by modulating p53 activity. Blood. 2017;130(24):2619–2630. doi: 10.1182/blood-2017-03-771386. 29084772 PMC5731083

[143] Heideman MR, Lancini C, Proost N, et al. Sin3a-associated Hdac1 and Hdac2 are essential for hematopoietic stem cell homeostasis and contribute differentially to hematopoiesis. Haematologica. 2014;99(8):1292–1303. doi: 10.3324/haematol.2013.092643. 24763403 PMC4116827

[144] Jian W, Yan BW, Huang SM, et al. Histone deacetylase 1 activates PU.1 gene transcription through regulating TAF9 deacetylation and transcription factor IID assembly. Faseb J. 2017;31(9):4104–4116. doi: 10.1096/fj.201700022R. 28572446 PMC5572695

[145] Vong P, Messaoudi K, Jankovsky N, et al. HDAC6 regulates human erythroid differentiation through modulation of JAK2 signalling. J Cell Mol Med. 2023;27(2):174–188. doi: 10.1111/jcmm.17559. 36578217 PMC9843532

[146] Yang C, Croteau S, Hardy P. Histone deacetylase (HDAC) 9: versatile biological functions and emerging roles in human cancer. Cell Oncol (Dordr). 2021;44(5):997–1017. doi: 10.1007/s13402-021-00626-9. 34318404 PMC8516780

[147] Barneda-Zahonero B, Collazo O, Azagra A, et al. The transcriptional repressor HDAC7 promotes apoptosis and c-Myc downregulation in particular types of leukemia and lymphoma. Cell Death Dis. 2015;6(2):e1635–e1635. doi: 10.1038/cddis.2014.594. 25675295 PMC4669785

[148] Yue LZ, Sharma V, Horvat NP, et al. HDAC11 deficiency disrupts oncogene-induced hematopoiesis in myeloproliferative neoplasms. Blood. 2020;135(3):191–207. doi: 10.1182/blood.2019895326. 31750881 PMC6966930

[149] Ginder GD, Whitters MJ, Pohlman JK. Activation of a chicken embryonic globin gene in adult erythroid-cells by 5-azacytidine and sodium-butyrate. Proc Natl Acad Sci USA. 1984;81(13):3954–3958. doi: 10.1073/pnas.81.13.3954. 6204332 PMC345346

[150] Perrine SP, Rudolph A, Faller DV, et al. Butyrate infusions in the ovine fetus delay the biologic clock for globin gene switching. Proc Natl Acad Sci USA. 1988;85(22):8540–8542. doi: 10.1073/pnas.85.22.8540. 2460870 PMC282494

[151] Perrine SP, Ginder GD, Faller DV, et al. A short-term trial of butyrate to stimulate fetal-globin gene-expression in the beta-globin disorders. N Engl J Med. 1993;328(2):81–86. doi: 10.1056/Nejm199301143280202. 7677966

[152] Perrine SP, Olivieri NF, Faller DV, et al. Butyrate derivatives – new agents for stimulating fetal globin production in the beta-globin disorders. Am J Pediat Hematol. 1994;16(1):67–71. 7508690

[153] Fathallah H, Weinberg RS, Galperin Y, et al. Role of epigenetic modifications in normal globin gene regulation and butyrate-mediated induction of fetal hemoglobin. Blood. 2007;110(9):3391–3397. doi: 10.1182/blood-2007-02-076091. 17638855 PMC2200921

[154] Bottardi S, Ross J, Bourgoin V, et al. Ikaros and GATA-1 combinatorial effect is required for silencing of human γ-globin genes. Mol Cell Biol. 2009;29(6):1526–1537. doi: 10.1128/Mcb.01523-08. 19114560 PMC2648246

[155] Zhao Q, Cumming H, Cerruti L, et al. Site-specific acetylation of the fetal globin activator NF-E4 prevents its ubiquitination and regulates its interaction with the histone deacetylase, HDAC1. J Biol Chem. 2004;279(40):41477–41486. doi: 10.1074/jbc.M405129200. 15273251

[156] Mankidy R, Faller DV, Mabaera R, et al. Short-chain fatty acids induce γ-globin gene expression by displacement of a HDAC3-NCoR repressor complex. Blood. 2006;108(9):3179–3186. doi: 10.1182/blood-2005-12-010934. 16849648 PMC1895523

[157] Muralidhar SA, Ramakrishnan V, Kalra IS, et al. Histone deacetylase 9 activates γ-globin gene expression in primary erythroid cells. J Biol Chem. 2011;286(3):2343–2353. doi: 10.1074/jbc.M110.115725. 21078662 PMC3023528

[158] Valerio DG, Xu HM, Eisold ME, et al. Histone acetyltransferase activity of MOF is required for adult but not early fetal hematopoiesis in mice. Blood. 2017;129(1):48–59. doi: 10.1182/blood-2016-05-714568. 27827827 PMC5216264

[159] Rodrigues CP, Akhtar A. Differential H4K16ac levels ensure a balance between quiescence and activation in hematopoietic stem cells. Sci Adv. 2021;7(32):eabi5987. doi: 10.1126/sciadv.abi5987. 34362741 PMC8346211

[160] Rodrigues CP, Herman JS, Herquel B, et al. Temporal expression of MOF acetyltransferase primes transcription factor networks for erythroid fate. Sci Adv. 2020;6(21):eaaz4815. doi: 10.1126/sciadv.aaz4815. 32671208 PMC7314555

[161] Grigoryan A, Guidi N, Senger K, et al. LaminA/C regulates epigenetic and chromatin architecture changes upon aging of hematopoietic stem cells. Genome Biol. 2018;19(1):189. doi: 10.1186/s13059-018-1557-3. 30404662 PMC6223039

[162] Hensold JO, Swerdlow PS, Housman DE. A transient increase in histone H2a ubiquitination is coincident with the onset of erythroleukemic cell-differentiation. Blood. 1988;71(4):1153–1156. doi: 10.1182/blood.V71.4.1153.1153. 2833326

[163] Kanda F, Sykes DE, Matsui S, et al. Isopeptidase has strict specificity for the chromatin protein A24 (Uh2a), a conjugate of histone and ubiquitin. J Cell Biol. 1984;99(4): a 135–A135.

[164] Nijnik A, Clare S, Hale C, et al. The critical role of histone H2A-deubiquitinase Mysm1 in hematopoiesis and lymphocyte differentiation. Blood. 2012;119(6):1370–1379. doi: 10.1182/blood-2011-05-352666. 22184403

[165] Lord KA, Creasy CL, King AG, et al. REDK, a novel human regulatory erythroid kinase. Blood. 2000;95(9):2838–2846. doi: 10.1182/blood.V95.9.2838.009k29_2838_2846. 10779429

[166] Jeffery NN, Davidson C, Peslak SA, et al. Histone H2A.X phosphorylation and caspase-initiated chromatin condensation in late-stage erythropoiesis. Epigenet Chromatin. 2021;14(1):37. doi: 10.1186/s13072-021-00408-5. PMC8325214 34330317

[167] Xie ZY, Dai JBA, Dai LZ, et al. Lysine succinylation and lysine malonylation in histones. Mol Cell Proteomics. 2012;11(5):100–107. doi: 10.1074/mcp.M111.015875. 22389435 PMC3418837

[168] Wang YG, Guo YR, Liu K, et al. KAT2A coupled with the α-KGDH complex acts as a histone H3 succinyltransferase. Nature. 2017;552(7684):273–277. doi: 10.1038/nature25003. 29211711 PMC5841452

[169] Hu B, Gong H, Nie L, et al. Lysine succinylation precisely controls normal erythropoiesis. Haematologica. 2025;110(2):397–413. doi: 10.3324/haematol.2024.285752. 39415677 PMC11788629

[170] Tang HR, An SM, Zhen HY, et al. Characterization of combinatorial histone modifications on lineage-affiliated genes during hematopoietic stem cell myeloid commitment. Acta Biochim Biophys Sin (Shanghai). 2014;46(10):894–901. doi: 10.1093/abbs/gmu078. 25205219

[171] Chaturvedi CP, Hosey AM, Palii C, et al. Dual role for the methyltransferase G9a in the maintenance of β-globin gene transcription in adult erythroid cells. Proc Natl Acad Sci USA. 2009;106(43):18303–18308. doi: 10.1073/pnas.0906769106. 19822740 PMC2775297

[172] Papadopoulos GL, Karkoulia E, Tsamardinos I, et al. GATA-1 genome-wide occupancy associates with distinct epigenetic profiles in mouse fetal liver erythropoiesis. Nucleic Acids Res. 2013;41(9):4938–4948. doi: 10.1093/nar/gkt167. 23519611 PMC3643580

[173] Popova EY, Krauss SW, Short SA, et al. Chromatin condensation in terminally differentiating mouse erythroblasts does not involve special architectural proteins but depends on histone deacetylation. Chromosome Res. 2009;17(1):47–64. doi: 10.1007/s10577-008-9005-y. 19172406 PMC2667965

[174] Okam MM, Esrick EB, Mandell E, et al. Phase 1/2 trial of vorinostat in patients with sickle cell disease who have not benefitted from hydroxyurea. Blood. 2015;125(23):3668–3669. doi: 10.1182/blood-2015-03-635391. 26045597 PMC4458806

[175] Junker LH, Li BAR, Zhu XG, et al. Novel histone deacetylase inhibitor CT-101 induces γ-globin gene expression in sickle erythroid progenitors with ­targeted epigenetic effects. Blood Cells Mol Dis. 2022;93:102626. doi: 10.1016/j.bcmd.2021.102626. 34856533 PMC9733664

[176] Atweh GF, Sutton M, Nassif I, et al. Sustained induction of fetal hemoglobin by pulse butyrate therapy in sickle cell disease. Blood. 1999;93(6):1790–1797. 10068649 PMC4269326

[177] Voskou S, Phylactides M, Afantitis A, et al. MS-275 chemical analogues promote hemoglobin production and erythroid differentiation of K562 cells. Hemoglobin. 2019;43(2):116–121. doi: 10.1080/03630269.2019.1626740. 31280628

[178] Mettananda S, Fisher CA, Sloane-Stanley JA, et al. Selective silencing of α-globin by the histone demethylase inhibitor IOX1: a potentially new pathway for treatment of β-thalassemia. Haematologica. 2017;102(3):E80–E84. doi: 10.3324/haematol.2016.155655. 27810991 PMC5394973

[179] Cui SY, Lim KC, Shi LH, et al. The LSD1 inhibitor RN-1 induces fetal hemoglobin synthesis and reduces disease pathology in sickle cell mice. Blood. 2015;126(3):386–396. doi: 10.1182/blood-2015-02-626259. 26031919 PMC4504950

[180] Bou-Fakhredin R, De Franceschi L, Motta I, et al. Pharmacological induction of fetal hemoglobin in β-thalassemia and sickle cell disease: an updated perspective. Pharmaceuticals-Base. 2022;15(6):753. doi: 10.3390/ph15060753. PMC9227505 35745672

[181] Nualkaew T, Khamphikham P, Pongpaksupasin P, et al. UNC0638 induces high levels of fetal hemoglobin expression in β-thalassemia/HbE erythroid progenitor cells. Ann Hematol. 2020;99(9):2027–2036. doi: 10.1007/s00277-020-04136-w. 32567028

