# Supplementary material for: Histone modifications in the regulation of erythropoiesis
Source: Ann Med. 2025 Apr 11;57(1):2490824. doi: 10.1080/07853890.2025.2490824 (PMC11995772; doi:10.1080/07853890.2025.2490824)
Supplement: Figure legend.docx [file IANN_A_2490824_SM7523.docx]

**Figure legends**

**Figure 1. The role of histone methylation in erythropoiesis.**

Erythropoiesis is the process of HSC differentiation into erythrocyte, which is mainly divided into three stages: the early erythropoiesis stage (light blue differentiation path), terminal erythroid differentiation stage (light orange differentiation path), and erythrocyte maturation stage (pink path). The upper part of the diagram shows the role of histone methylation and related modifying enzymes (numbers representing methylation sites, R representing arginine residues, K representing lysine residues, protein-directed methylation indicating that the protein is methyltransferases, and methylation-directed protein indicating that the protein is demethylase) in early erythropoiesis. It is mainly involved in regulating the function of hematopoietic stem progenitor cells and the growth and development of erythroid progenitor cells. The lower part of the diagram shows the role of histone methylation and related modification enzymes in the terminal erythroid differentiation, which mainly regulates chromatin condensation, enucleation and globin expression in erythroblast.

**Figure 2. The role of histone acetylation in erythropoiesis.**

Erythropoiesis is the process of HSC differentiation into erythrocyte, which is mainly divided into three stages: the early erythropoiesis stage (light blue differentiation path), terminal erythroid differentiation stage (light orange differentiation path), and erythrocyte maturation stage (pink path). The left part of the diagram shows the role of histone acetylation and related modifying enzymes (numbers representing methylation sites, K representing lysine residues, protein-directed acetylation indicating that the protein is acetyltransferases, and acetylation-directed protein indicating that the protein is deacetylases) in early erythropoiesis. It is mainly involved in regulating the function of hematopoietic stem progenitor cells and the development of erythroid progenitor cells. The right part of the diagram shows the role of histone acetylation and related modification enzymes in the terminal erythroid differentiation, which mainly regulates chromatin condensation, enucleation and globin expression in erythroblast.

**Figure 3. Schematic diagram of histone modification sites acting in erythropoiesis.**

The left side of the diagram shows the different cell lines during erythropoiesis including HSC, CFU-E, polychromatic erythroblast, reticulocyte, and so on. The right side of the diagram shows the main modification sites of H3 and H4 histones, and the information of the modification sites is marked on the N-terminal of H3 and H4 histones. The numbers show the position of each retouching. The letters indicate the amino acid or type of modification at each modification site: K representing lysine residues, R representing arginine residues, M representing methylated modification, and A representing acetylated modification. In addition, the diagram also shows that different modification sites and types are involved in the differentiation process of different stages of erythropoiesis by altering the expression of key genes such as Runx1, GATA1, etc.
